# Replicon particle vaccination induces non-neutralizing anti-nucleoprotein antibody-mediated control of Crimean-Congo hemorrhagic fever virus

**DOI:** 10.1038/s41541-024-00877-1

**Published:** 2024-05-23

**Authors:** Teresa E. Sorvillo, Elif Karaaslan, Florine E. M. Scholte, Stephen R. Welch, JoAnn D. Coleman-McCray, Sarah C. Genzer, Jana M. Ritter, Heather M. Hayes, Shilpi Jain, Scott D. Pegan, Éric Bergeron, Joel M. Montgomery, Christina F. Spiropoulou, Jessica R. Spengler

**Affiliations:** 1 Viral Special Pathogens Branch, Division of High Consequence Pathogens and Pathology, Centers for Disease Control and Prevention, Atlanta, GA USA; 2 Infectious Disease Department, CDC Foundation, Atlanta, GA USA; 3 Division of Biomedical Sciences, University of California Riverside, Riverside, CA USA; 4 Infectious Diseases Pathology Branch, Division of High Consequence Pathogens and Pathology, Centers for Disease Control and Prevention, Atlanta, GA USA

**Keywords:** Vaccines, Virology

## Abstract

Crimean-Congo hemorrhagic fever virus (CCHFV) can cause severe human disease and is considered a WHO priority pathogen due to the lack of efficacious vaccines and antivirals. A CCHF virus replicon particle (VRP) has previously shown protective efficacy in a lethal Ifnar^-/-^ mouse model when administered as a single dose at least 3 days prior to challenge. Here, we determine that non-specific immune responses are not sufficient to confer short-term protection, since Lassa virus VRP vaccination 3 days prior to CCHFV challenge was not protective. We also investigate how CCHF VRP vaccination confers protective efficacy by examining viral kinetics, histopathology, clinical analytes and immunity early after challenge (3 and 6 days post infection) and compare to unvaccinated controls. We characterize how these effects differ based on vaccination period and correspond to previously reported CCHF VRP-mediated protection. Vaccinating Ifnar^-/-^ mice with CCHF VRP 28, 14, 7, or 3 days prior to challenge, all known to confer complete protection, significantly reduced CCHFV viral load, mucosal shedding, and markers of clinical disease, with greater reductions associated with longer vaccination periods. Interestingly, there were no significant differences in innate immune responses, T cell activation, or antibody titers after challenge between groups of mice vaccinated a week or more before challenge, but higher anti-NP antibody avidity and effector function (ADCD) were positively associated with longer vaccination periods. These findings support the importance of antibody-mediated responses in VRP vaccine-mediated protection against CCHFV infection.

## Introduction

Crimean-Congo hemorrhagic fever (CCHF) virus (CCHFV; family *Nairoviridae*, genus *Orthonairovirus*) is the most geographically widespread tick-borne virus, endemic to large areas of Asia, Africa, and Europe, with a range that continues to expand^1^. Recent large-scale outbreaks in Iraq and Georgia suggest that the incidence of disease is also increasing^2–4^. Data on the annual global incidence of CCHF are limited and the true burden of disease is likely underreported^5,6^. Regions in which the virus is highly endemic, like Turkey, report several hundred cases per year^7^ despite detecting only the subset of cases that require hospitalization. CCHFV is primarily transmitted via the bite of *Hyalomma* species ticks or through exposure to the blood of infected livestock during slaughter practices^8^. Nosocomial infections have been reported infrequently but can be associated with high case fatality rates^9–12^. Due to its expanding endemic range, potential for high fatality rates, and lack of available countermeasures, including safe and efficacious FDA-licensed vaccines, CCHFV is considered a World Health Organization (WHO) priority pathogen^13^.

CCHFV infection causes a wide spectrum of human clinical disease, ranging from mild febrile illness to severe hemorrhagic disease with case fatality rates of 10–40%^8^. Apart from suckling mice, animal models of lethal CCHFV infection have only been described as recently as 2010 and require either virus adaptation or suppression of type I interferon (IFN) responses (e.g., Ifnar^-/-^, Ifnagr^-/-^, or Stat-1^-/-^ mice)^14–16^. Although these models have inherent limitations, they recapitulate important features of severe human CCHF, like liver pathology, spleen-associated lymphocyte depletion, and immune dysregulation including elevated levels of the inflammatory cytokines/chemokines IL-6, TNF-alpha, IL-18, and CCL2^17–20^.

Numerous experimental CCHF vaccine candidates have been described, but none have received FDA approval. Several important factors require consideration in the development of candidate vaccines, including the fact that CCHFV is more genetically diverse than other arboviruses, containing as many as 7 recognized clades. CCHFV is a negative-sense, single-stranded RNA virus containing 3 genomic segments, small (S), medium (M), and large (L), which encode a nucleoprotein (NP), glycoprotein precursor (GPC), and RNA-dependent RNA polymerase (L), respectively. Genomic sequence divergence between clades is 20%, 31%, and 22% in the S, M, and L segments, respectively^8,21,22^. Ideally, a vaccine would demonstrate heterologous protection against several viral clades. Additionally, a single-dose vaccine would be highly advantageous given that, in many endemic countries, persons most at risk for infection tend to live in rural locales with less immediate access to preventative healthcare^23–25^. While promising vaccine candidates have been described, few have demonstrated heterologous protection, and most require several doses to generate protective efficacy in vivo.

To date, only 3 vaccine candidates have demonstrated some or all of these important features. First, a vesicular stomatitis virus-based viral vector vaccine containing the CCHFV strain IbAr10200 (clade Africa 3) glycoprotein is fully protective after single-dose vaccination against strain Turkey-200406546 (Turkey04; clade Europe 1) challenge^26^. However, no data have been reported regarding efficacy against viruses from other clades^26^. A DNA vaccine using the IbAr10200 sequence (M segment) has been shown to protect 100% and 80% of animals against homologous and heterologous (strain Afg09-2990, clade Asia 1) challenge, respectively; however, this vaccine requires a 3-dose regimen^27^. Lastly, a CCHFV viral replicon particle (VRP) has shown 100% protective efficacy against homologous and heterologous CCHFV challenge, including against viruses from 3 clades (Africa 3 [IbAr10200], Europe 1 [Turkey04], and Asia 1 [Oman-97]) when given as a single dose to Ifnar^-/-^ mice 1 month prior to infection^28,29^. This broad protection may be due to the chimeric nature of the VRP, which is generated from the IbAr10200 reverse genetics system but carries the Oman-98 strain (clade Asia 1) glycoprotein on its envelope to enhance entry into target cells^29^. The VRP mimics the morphology of a CCHF viral particle but lacks the M genome segment, which results in authentic virion entry but only a single round of replication, making it immunogenic but apathogenic^29^. Additionally, the VRP can protect mice from lethal outcome when administered to Ifnar^-/-^ mice as a short-course single dose, 3 days before challenge^30^.

The mechanisms by which CCHF VRP-mediated protection is achieved, and how these mechanisms vary based on the timing of vaccination, are currently unknown. Here, to advance our previously published efficacy studies, we conducted a series of serial euthanasia experiments to investigate how the timing of CCHF VRP vaccination affects viral replication and dissemination, tissue pathology, clinical analytes, and immune responses after CCHFV challenge. Importantly, we used the same animal model (Ifnar^-/-^ mice) from prior efficacy studies in which vaccination 28, 14, 7, or 3 days prior to challenge was shown to confer complete protection. We also explored the roles of CCHF VRP platform-specific features in vaccine efficacy by using a non-specific Lassa virus (LASV) VRP vaccine to investigate the importance of antigen specificity in short-course protection, and a UV-inactivated vaccine preparation to investigate the necessity of single-round replication for efficacy.

## Results

### CCHF VRP platform requires antigen specificity and single-round replication for efficacy

**Fig. 1:**
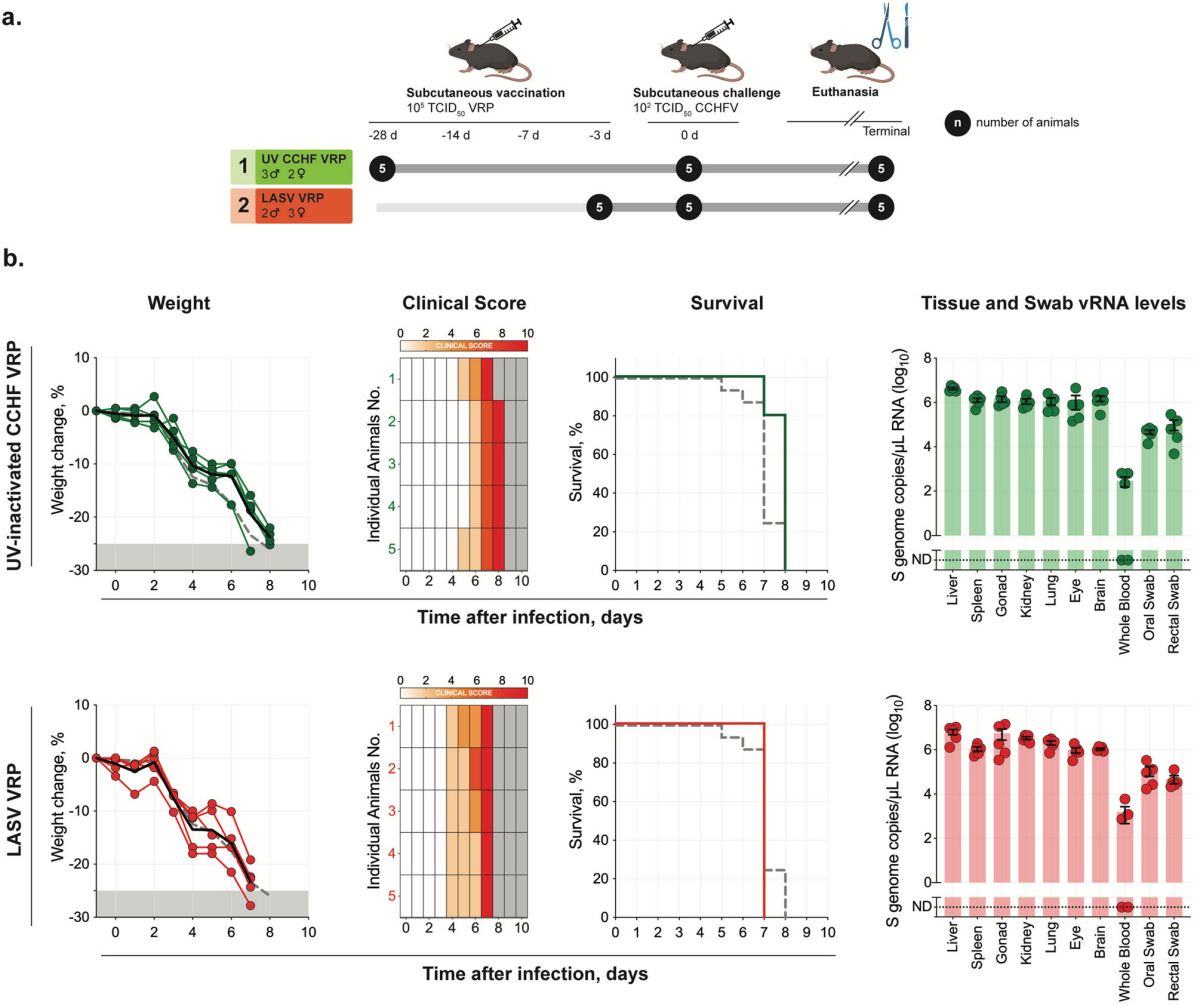
CCHF VRP platform requires single-round replication and antigen specificity for efficacy. **a** Study timeline: Ifnar^-/-^ mice were vaccinated with a UV-inactivated CCHF VRP inoculum (*n* = 5) 28 days prior to challenge or with a non-specific LASV VRP inoculum (*n* = 5) 3 days prior to challenge. Vaccines were administered subcutaneously (SC) with a dose of 1.00 × 10^5^ TCID_50_/animal. All mice were challenged SC with 100 TCID_50_ CCHFV strain Turkey04 and followed to their terminal endpoint (endpoint criteria described in Methods). After challenge, **b** weight loss (% change from baseline at -1 dpi), clinical score (described in Methods), and survival were recorded daily for each animal. Weight and survival data from historical controls are represented by dashed gray lines. Tissues (liver, spleen, ovary/testis, kidney, lung, heart, eye, brain, whole blood) and oropharyngeal and rectal swabs were collected from terminal animals and evaluated by RT-qPCR to quantify levels of CCHFV RNA using a primer/probe set specific for the NP ORF of the CCHFV S gene segment. Individual animals are represented. Bars and error bars indicate mean ± standard error of the mean (SEM).

We previously showed that the CCHF VRP fully protects Ifnar^-/-^ mice from lethal CCHFV infection when administered in as few as 3 days prior to challenge (short-course vaccination)^30^. Data derived from other vaccines demonstrate that rapid protection after vaccination can sometimes be attributed to innate immune responses alone^31,32^. We therefore first investigated whether CCHF VRP short-course protection could result from non-specific antiviral immune responses induced early post vaccination. Groups of mice (*n* = 5) were vaccinated with a similar VRP platform based on a distantly related virus, LASV (described by Kainulainen *et al*., 2018), and challenged 3 days later with CCHFV to determine if non-specific immune response contributed to protection (Fig. 1a)^30,33^.

Secondly, a distinctive characteristic of the CCHF VRP vaccine is its ability to undergo a single round of replication/transcription upon cell entry; however, the importance of this feature in vaccine efficacy is unclear. To determine the necessity of single-round replication in CCHF VRP efficacy, a UV-inactivated VRP inoculum was administered to mice (*n* = 5) 28 days prior to challenge (-28D) (Fig. 1a).

Animals from both LASV VRP and UV-inactivated CCHF VRP vaccine cohorts were subcutaneously (SC) challenged with heterologous CCHFV strain Turkey04 (100 TCID_50_), monitored daily for signs of clinical disease and weight loss, and followed to their terminal endpoint to determine survival outcomes. Mice vaccinated with either inoculum uniformly succumbed to disease 7–8 days post infection (dpi) with no delay in time to death compared with historical non-vaccinated controls^28,30^; weight loss and signs of clinical disease began 4 (LASV VRP) or 5−6 dpi (UV-inactivated CCHF VRP) (Fig. 1b). CCHF viral RNA (vRNA) titers in tissues, whole blood, and swabs were also comparable to those seen in historical control animals at the time of euthanasia (Fig. 1b)^28,30^. These data demonstrate the necessity of single-round replication in CCHF VRP efficacy and of antigen specificity for rapid short-course protection.

### CCHF VRP vaccination reduces potential for CCHFV transmission

Previous studies to assess the effects of CCHF VRP vaccination on disease outcome have followed animals until they met end-point criteria due to clinical disease or until the end of the predetermined study period^28–30^. These studies demonstrated that vaccination prevented clinical disease, including weight loss, in animals vaccinated 28, 14, or 7 days prior to challenge (-28D, -14D, and -7D, respectively). Animals vaccinated 3 days prior to challenge (-3D) developed mild, transient weight loss but no other clinical signs. These studies also indicated that while vaccination prevents lethality and reduces clinical disease, protection is non-sterilizing^28,29^. We therefore sought to determine whether vaccination reduces the potential for virus transmission at serial timepoints after challenge. Studies in humans and non-human primates (NHP) have shown that CCHF vRNA can be detected from both oral and rectal swab samples via RT-qPCR^34,35^. While human-to-human transmission of CCHFV is thought to be infrequent, it does occur in nosocomial settings and these clusters of cases tend to result in high case fatality rates, making mucosal shedding/transmission an important metric for consideration in CCHFV vaccine development^12,36–38^.

**Fig. 2:**
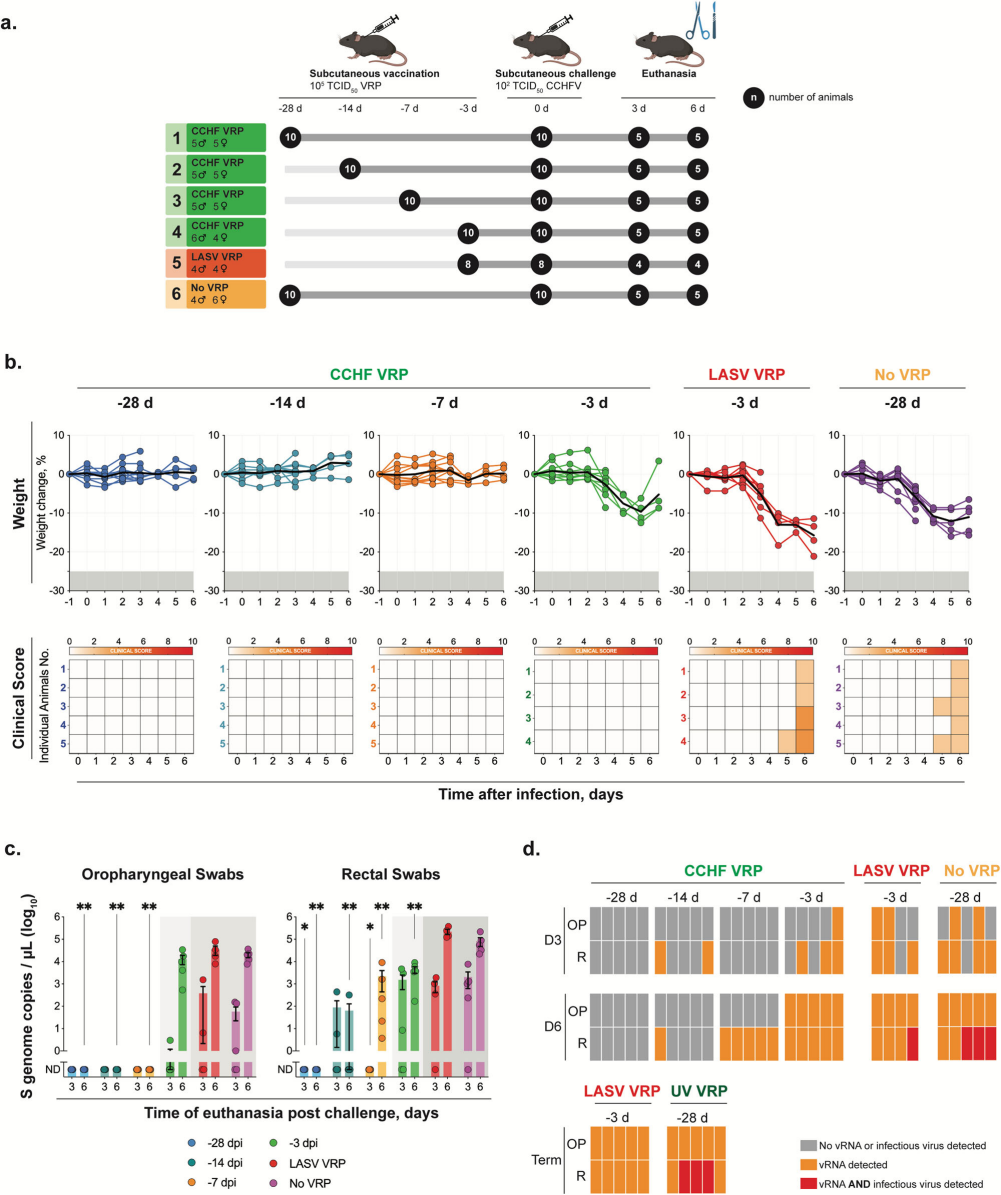
CCHF VRP vaccination reduces potential for virus transmission after infection. **a** Study timeline for vaccination, challenge, and serial euthanasia. Cohorts of Ifnar^-/-^ mice (*n* = 8–10) were vaccinated SC with CCHF VRP or LASV VRP (both at 1.00 × 10^5^ TCID_50_), or left unvaccinated (no VRP, given DMEM alone) 28, 14, 7, or 3 days (-28D, -14D, -7D, -3D) prior to challenge with lethal CCHFV Turkey04 (100 TCID_50_, SC). Cohorts from each group (*n* = 4–5) were serially euthanized 3 or 6 dpi. **b** After challenge, weight loss (% change from baseline at -1 dpi) and clinical score (described in Methods) were recorded daily for each animal. Paired oropharyngeal (OP) and rectal (R) swabs were collected from all animals at the time of euthanasia and used for **c** viral RNA quantification (individual animals are represented, bars and error bars indicate mean ± SEM) or **d** CCHFV isolation (individual animals are represented). vRNA was quantified by RT-qPCR using a primer/probe set specific for the NP ORF of the CCHFV S gene segment. CCHFV was isolated through inoculation, fixation, and immunostaining of BSR-T7/5 cells. Statistics for each vaccine group were calculated as significant change compared to unvaccinated control animals (No VRP, given DMEM alone) at equivalent timepoint (3 or 6 dpi) using multiple two-tailed *t*-tests (Mann-Whitney). Only statistically significant results are reported; *p < 0.5; **p < 0.01 (Supplementary Table 1).

Groups of Ifnar^-/-^ mice (*n* = 8–10) were vaccinated subcutaneously (SC) with CCHF VRP or LASV VRP (both at 1.00 × 10^5^ TCID_50_), or remained unvaccinated (no VRP, given DMEM alone) -28D, -14D, -7D, or -3D before a lethal SC challenge with CCHFV Turkey04 (100 TCID_50_) (Fig. 2a). Cohorts from each group (*n* = 4–5) were then serially euthanized at 3 or 6 dpi. All animals were monitored daily for weight loss and signs of clinical disease after challenge. Consistent with our previous findings, CCHF VRP vaccination 28, 14, or 7 days prior to challenge prevented clinical signs and weight loss in infected animals. Maximum average weight loss from baseline in -28D, -14D, and -7D groups was -1.5%, -0.9%, and -1.7%, respectively, percentages that are within normal ranges of weight fluctuation for healthy mice^39^. Mice in the -3D group lost weight (maximum average: 12.7% from baseline) between 3 and 5 dpi, but by day 6 had begun to recover it (Fig. 2b). Control animals receiving DMEM alone began to show signs of clinical disease 4–6 dpi, including weight loss (maximum average for DMEM group: -12.4%; LASV VRP group: -14.6%), hunched posture, rough coat, and hypoactivity, with no signs of improvement by day 6 (Fig. 2b).

To determine transmission potential, paired oropharyngeal and rectal swabs were collected for virus isolation and RT-qPCR analyses from all animals at the time of euthanasia (3 or 6 dpi). In the -28D group, vRNA was undetectable in both oral and rectal swabs from all animals. In -14D and -7D animals, vRNA was undetectable in oral swabs and significantly reduced in rectal swabs compared to unvaccinated controls (Fig. 2c). Oral swabs from short-course vaccinated (-3D) animals were not significantly different from control animals but rectal swabs were significantly lower at 6 dpi. We then investigated whether infectious virus could be isolated from RT-qPCR-positive swabs. No infectious virus could be isolated from oral swabs, including from control animals with the highest vRNA levels (Fig. 2d). Infectious virus was isolated from a subset of rectal swabs from control animals (LASV VRP and mock vaccinated) but not from any CCHF VRP-vaccinated animals (including -3D group) (Fig. 2d). These findings were not surprising because vRNA levels were, on average, 10-fold higher in rectal than oral swabs. While infectious virus could be isolated from rectal swabs of control animals, the levels were below the limit of detection for TCID_50_ quantification in all but one animal, which was mock vaccinated (DMEM only) and euthanized 6 dpi (21.7 TCID_50_/mL). Importantly, the lack of virus isolated from all vaccinated groups supports the RT-qPCR findings of decreased viral load in these animals compared to controls, suggesting lowered transmission risk.

### Reduction of clinical disease after CCHF VRP vaccination is the result of lower viral load and preserved liver function

**Fig. 3:**
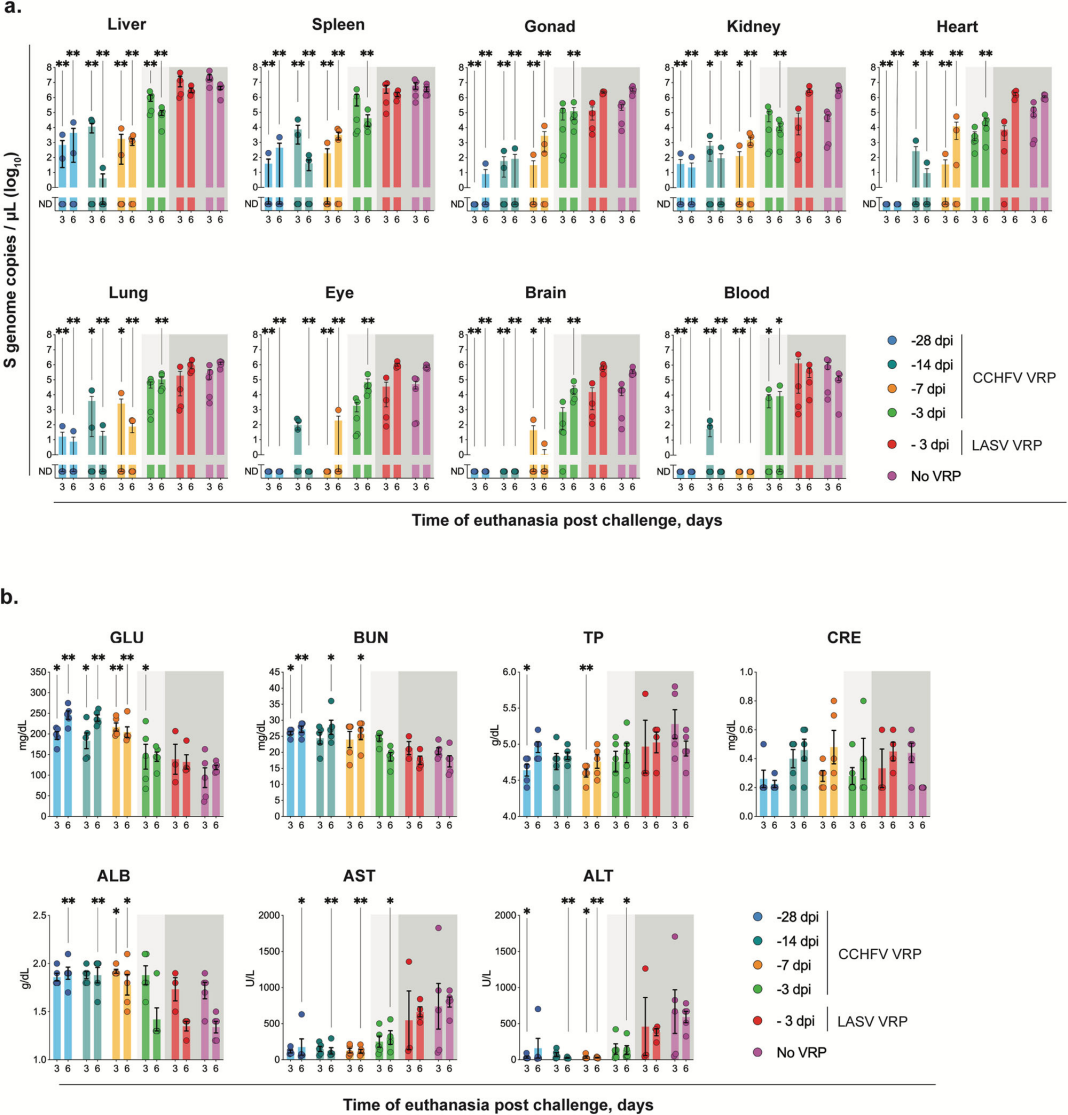
CCHF VRP reduces clinical disease by controlling viral load and improving liver function. **a** Tissues including liver, spleen, ovary/testis (gonad), kidney, lung, heart, eye, brain, and whole blood were collected from mice in all vaccine cohorts at the time of euthanasia 3 or 6 days post infection (dpi). Viral RNA was quantified via RT-qPCR using primers/probe specific for the NP ORF of the CCHFV S gene segment. **b** Clinical chemistry analytes from each animal were assessed using whole blood collected peri-mortem (intracardiac bleed) in lithium heparin and analyzed via the General Chemistry 13 Panel on the Piccolo Xpress analyzer. GLU glucose, BUN blood urea nitrogen, ALB albumin, ALT alanine aminotransferase, AST aspartate aminotransferase, TP total protein, CRE creatinine. Viral load and clinical chemistry statistics for each vaccine group were calculated as significant change compared to unvaccinated control animals (No VRP, given DMEM alone) at equivalent timepoint (3 or 6 dpi) using multiple two-tailed *I*-tests (Mann-Whitney). Only statistically significant results are reported; *p < 0.5; **p < 0.01 (Supplementary Tables 1-2). Individual animals are represented. Bars and error bars indicate mean ± SEM.

We observed that mice vaccinated further from the time of challenge had less pronounced clinical disease and lower levels of vRNA detectable in swabs, suggesting that clinical disease correlates with viral load. Previous studies have shown that vRNA can still be detected in surviving CCHF VRP-vaccinated mice at low levels 21 days after challenge^22^; however, the kinetics of viral replication in these animals has never been investigated. Here, we evaluated the quantity of vRNA in whole blood and tissues, including in the liver, spleen, kidney, ovary/testis, heart, lung, eye, and brain at early (3 dpi) and late (6 dpi) timepoints after challenge. We found that, overall, CCHF VRP-vaccinated animals had reduced viral loads compared with control cohorts, and that this reduction was most pronounced in animals vaccinated 28 days prior to infection (Fig. 3a). Levels of vRNA in tissues became incrementally lower and detected in fewer animals as the vaccine was administered further from the date of challenge (Fig. 3a). In the -28D cohort, vRNA was detected in 56% (5 of 9) of assessed tissues, whereas vRNA was detected in 89% (8 of 9) of tissue samples from -14D and -7D cohorts. Notably, vaccination 2 weeks prior to challenge or earlier resulted in undetectable vRNA in the brain post infection (Fig. 3a). Animals in the -3D cohort had higher levels of viral replication than other vaccine cohorts, but peak vRNA titers remained lower than those in control animals. Importantly, we observed that control of virus replication was already evident by 3 dpi in all vaccine cohorts, including -3D.

To determine how viral load contributes to clinical disease, we analyzed clinical chemistry analytes present in whole blood. At 6 dpi, we saw significantly lower liver enzyme values (alanine aminotransferase [ALT] and aspartate aminotransferase [AST]) in CCHF VRP-vaccinated animals than in those that received DMEM (Fig. 3b). Within vaccine cohorts, animals vaccinated -3D had the highest overall AST and ALT values, but these were still significantly lower than in unvaccinated, infected controls at 6 dpi. Glucose, blood urea nitrogen, and albumin were significantly lower in control animals than in -28D, -14D, and -7D CCHF VRP-vaccinated groups, likely indicating reduced food intake in animals with clinical symptoms (Fig. 3b). This trend was not observed in short-course vaccinated animals which developed mild clinical disease (Fig. 3b).

### Vaccination period >1 week significantly reduces distribution of viral antigen and associated tissue pathology

**Fig. 4:**
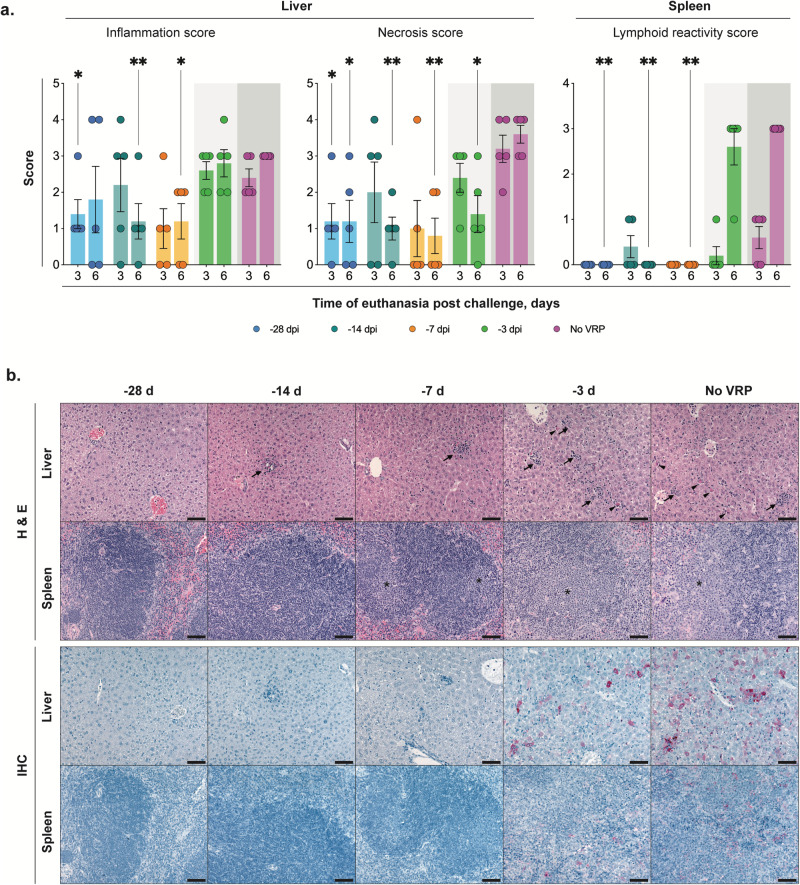
Vaccination reduces pathology and CCHFV antigen in liver and spleen. **a** Mean hepatic inflammation and necrosis and splenic lymphoid reactivity scores (0–4) (error bars represent standard deviation). Mean liver inflammation and necrosis scores were reduced with vaccination 1 week or more prior to inoculation, compared to short-course (-3D) or unvaccinated controls (no VRP, given DMEM alone). Mean liver inflammation scores were similar for short-course and unvaccinated animals at both 3 and 6 days post infection (dpi), but mean liver necrosis score decreased from 3 to 6 dpi in -3D animals and increased from 3 to 6 dpi in unvaccinated animals. Consistent lymphoid reactivity was present only in -3D and unvaccinated animals and increased from 3 to 6 dpi. Individual animals are represented. Bars and error bars indicate mean ± SEM. **b** Liver and spleen pathology (top two rows) and CCHFV antigen detection by immunohistochemistry (bottom two rows) at 6 dpi. No or rare small foci of inflammation and hepatocyte necrosis (arrows) were present with vaccine administration 7 or more days prior to inoculation. Livers in -3D and unvaccinated animals both have prominent inflammation and necrotic hepatocytes (arrowheads). Spleens from -28D- and -14D-vaccinated animals showed non-reactive follicles, while -7D-vaccinated animals showed mild reactivity, and spleens from -3D-vaccinated and unvaccinated mice showed similar, marked lymphoid reactivity characterized by follicular expansion by lymphoblasts and prominent plasma cells (*). Immunohistochemistry for CCHFV shows immunostaining (red) of necrotic hepatocytes in -3D and unvaccinated livers; more numerous, confluent clusters of hepatocytes were stained in livers of unvaccinated animals than in short-course vaccinees. Scattered staining of histiocytes was present in a -3D spleen and more prevalent in the spleen of an unvaccinated animal. No immunostaining was seen in livers or spleens from animals vaccinated 1 week or more prior to inoculation. Original magnifications 20 ×, scale bars are 50 μM. Top two rows: hematoxylin-eosin (H&E) stain; bottom two rows: CCHF immunohistochemistry (IHC) with Fast Red chromogen.

Brain, eye, heart, lung, liver, kidney, spleen, pancreas, trachea, esophagus, mediastinal lymph node, thymus, adrenal gland, bladder, stomach, intestine, mesenteric lymph node, and reproductive tissues were evaluated by histopathology 3 and 6 dpi in all vaccine groups and unvaccinated controls. Significant CCHFV-associated tissue pathology was present in liver and lymphoid tissues, and severity generally correlated inversely with the interval between vaccination and CCHFV inoculation (Fig. 4a and b). Liver changes included single-cell and confluent hepatocellular necrosis accompanied by mixed lobular inflammation, Kupffer cell reactivity, and intravascular leukocytosis. Hepatic inflammation and necrosis were scored semi-quantitatively on a scale of 0–4 (Fig. 4a). Mean scores for both were higher in tissues from -3D animals than in animals vaccinated -7D or earlier. Levels of inflammation and necrosis were similar in -3D and unvaccinated (given DMEM alone) vaccine cohorts at 3 dpi. Importantly, we found that from 3 to 6 dpi the average necrosis score in unvaccinated animals had increased while it had decreased in -3D vaccinees; inflammation scores remained similar for both groups at 6 dpi (Fig. 4a). Spleen and multiple lymph nodes (most often mediastinal, pancreatic, perirenal, and mesenteric) showed changes of reactive lymphoid hyperplasia, with follicular expansion by lymphoblasts and plasma cells; spleens also had extramedullary hematopoiesis. Lymphoid reactivity was only consistently evident in tissues of -3D and unvaccinated cohorts and was minimal at 3 dpi but prominent at 6 dpi in both groups (Fig. 4a).

**Fig. 5:**
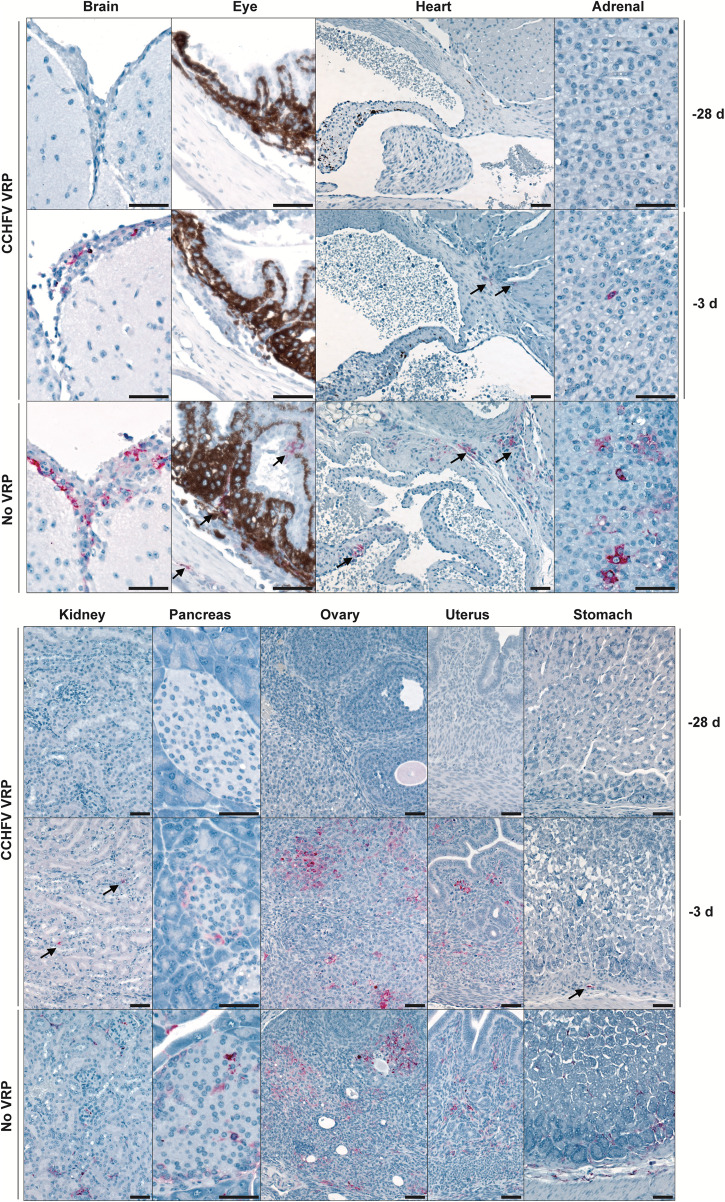
Disseminated CCHFV antigen is present in tissues with minimal or no pathology in short-course-vaccinated (-3D) and unvaccinated animals at 6 dpi. Top row: -28D vaccination; middle row: short-course (-3D) vaccination; bottom row: unvaccinated. In unvaccinated (no VRP, given DMEM alone) and short-course-vaccinated animals, CCHFV antigen (red; arrows) was present in scattered cells within the leptomeninges of the brain, ciliary body of the eye, valvular stroma of the heart, adrenal cortex, renal interstitium, pancreatic islets, ovarian follicles and stroma, uterine endometrial stroma, and gastric lamina propria and submucosa/serosa. No immunostaining was seen in the same tissue types from -28D vaccinated animals. Original magnifications: 20 × (heart, kidney, ovary, uterus, stomach); 40 × (brain, eye, pancreas, adrenal); scale bars are 50 μM. CCHFV immunohistochemistry with Fast Red chromogen.

Immunohistochemistry was performed 6 dpi to assess relative quantity and distribution of viral antigens in tissues in animals from all vaccine groups (Figs. 4b and 5). Antigen was almost exclusively detected in unvaccinated and -3D cohorts; antigen immunostaining correlated with extent of hepatocyte necrosis in liver and degree of lymphoid reactivity in spleen (Fig. 4b). In liver, staining colocalized to necrotic and intact hepatocytes, Kupffer cells, and, rarely, to endothelial cells and intravascular leukocytes. In spleen and lymph nodes, staining was within histiocytes. In unvaccinated and -3D vaccine cohorts, CCHFV immunostaining was also observed in other tissues with minimal lymphocytic infiltrates or without overt tissue pathology (Fig. 5). Such immunostaining was seen in leptomeninges of the brain; ciliary body, trabecular network, and endothelial cells of the corneoscleral junction of the eye; cardiac valvular endothelial and stromal cells and rare inflammatory cells at the base of the heart; adrenal cortex and medulla; renal interstitium, vascular endothelial cells, and glomeruli; pancreatic islets and interstitial stromal cells; ovarian follicles and stroma; uterine endometrial and oviductal stroma; testicular, epididymal, accessory sex gland, and urinary bladder stroma; stromal cells of the esophageal and gastrointestinal lamina propria and submucosa; and scattered interstitial, endothelial, and intravascular cells in the lung. Antigen was not detected in any tissues from animals vaccinated -7D, -14D, or -28D, except in two -28D animals with rare staining of individual hepatocytes.

Overall, pathology and IHC findings support other data from our study, indicating that morbidity is associated with hepatic necrosis due to direct viral infection of hepatocytes, which is largely prevented in animals vaccinated 7 or more days prior to infection, and occurs but is transient and resolving by 6 dpi after short-course vaccination. Lymphoid reactivity is similarly prevented by vaccination 7 or more days prior to infection but is sustained 6 dpi in short-course-vaccinated or unvaccinated animals. Investigations of later time points in future studies may provide insights into the timeframe of the resolution of reactive lymphoid changes.

### Anti-NP CCHFV antibodies are detectable early after challenge in short-course-vaccinated animals

**Fig. 6:**
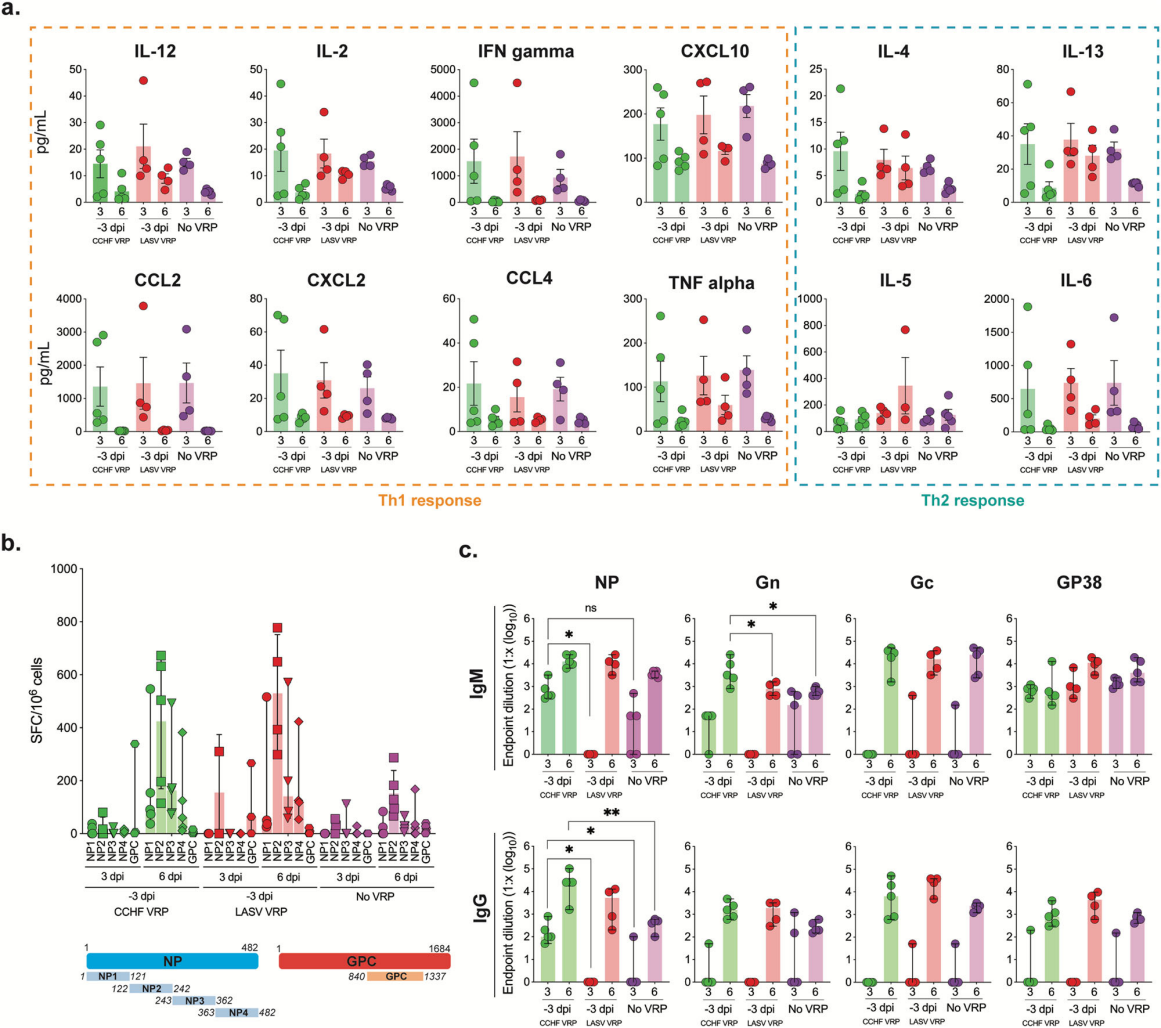
Anti-NP CCHFV antibodies are present early after challenge in short-course-vaccinated animals. **a** Cytokine/chemokine responses in mice vaccinated with CCHF VRP, LASV VRP, or left unvaccinated (no VRP, given DMEM alone) 3 days prior to challenge and euthanized 3 or 6 days post infection (dpi) were analyzed using the ProcartaPlex Mouse Th1/Th2 Cytokine and Chemokine panel and 25 µL mouse plasma. **b** CCHFV-specific T-cell responses were evaluated using IFN-gamma ELISpot assay. Peptides covering the CCHFV IbAr10200 NP or Oman-98 GPC NSm-Gc domain (numbers represent aa positions) were used to stimulate splenocytes harvested from vaccinated animals. Data are reported as the number of spot-forming cells (SPC)/1.00 x 10^6^ cells. **c** CCHFV-specific NP, Gn, Gc, and GP38 antibody responses (IgM and IgG) were evaluated via ELISA and are reported as endpoint dilution titers. Antibody titers, T-cell responses, and cytokine/chemokine levels were compared statistically between vaccine groups by timepoint (3 or 6 dpi) using multiple two-tailed *t*-tests (Mann-Whitney) and only statistically significant results are reported; *p < 0.5, **p < 0.01. Individual animals are represented. Bars and error bars indicate mean ± SEM.

We demonstrated that non-specific LASV VRP vaccination was insufficient to confer rapid protection against CCHFV, suggesting that virus-specific adaptive responses may be necessary for efficacy. To verify that these differences in outcome were not due to differences in the induction of early non-specific immune responses, we analyzed levels of Th1/Th2 cytokine/chemokines in plasma (Fig. 6a). Animals vaccinated with CCHF VRP 3 days prior to challenge displayed profiles of immune activation that were not significantly different at 3 or 6 dpi from those seen in LASV VRP- and DMEM-vaccinated animals (Fig. 6a), confirming that these specific innate responses do not confer protection.

To determine if CCHFV-specific adaptive immune responses could be detected in short-course-vaccinated animals, T-cell responses against CCHFV NP and GPC peptides were evaluated via IFN-gamma ELISpot using splenocytes isolated from mice 3 or 6 dpi. Short-course CCHF VRP-vaccinated mice showed similar T-cell activation to LASV VRP-vaccinated animals (Fig. 6b), suggesting these responses also may not be critical for CCHF VRP-mediated protective efficacy after challenge.

CCHFV IgM and IgG antibody responses were then assessed in all animals via ELISA; responses against NP and GPC-derived Gn, Gc, and GP38 viral proteins were all measured. Gn, Gc, and GP38 antibody titers were not significantly different between short-course CCHF VRP and LASV VRP-vaccinated groups early after infection (3 dpi) (Fig. 6c). Neutralizing antibody titers from plasma were also assessed and found to be low or undetectable in most animals 3 and 6 dpi (Supplementary Fig. 1). Interestingly, in -3D CCHF VRP-vaccinated animals, we found significantly higher anti-NP IgM titers 3 dpi than in LASV VRP-vaccinated animals. Additionally, we found that these animals had detectable, and significantly higher, anti-NP IgG titers than LASV VRP or DMEM control groups 3 dpi (Fig. 6c). While we were not able to show notable differences in innate or T-cell-mediated immune activation between CCHF and LASV VRP vaccine groups after challenge, we did detect higher levels of total anti-NP IgM and IgG titers in the CCHF VRP group by 3 dpi, indicating that these responses may be important for conferring rapid vaccine-mediated protection.

### Early control of virus replication is associated with presence of anti-NP antibodies rather than innate or cellular immune responses

**Fig. 7:**
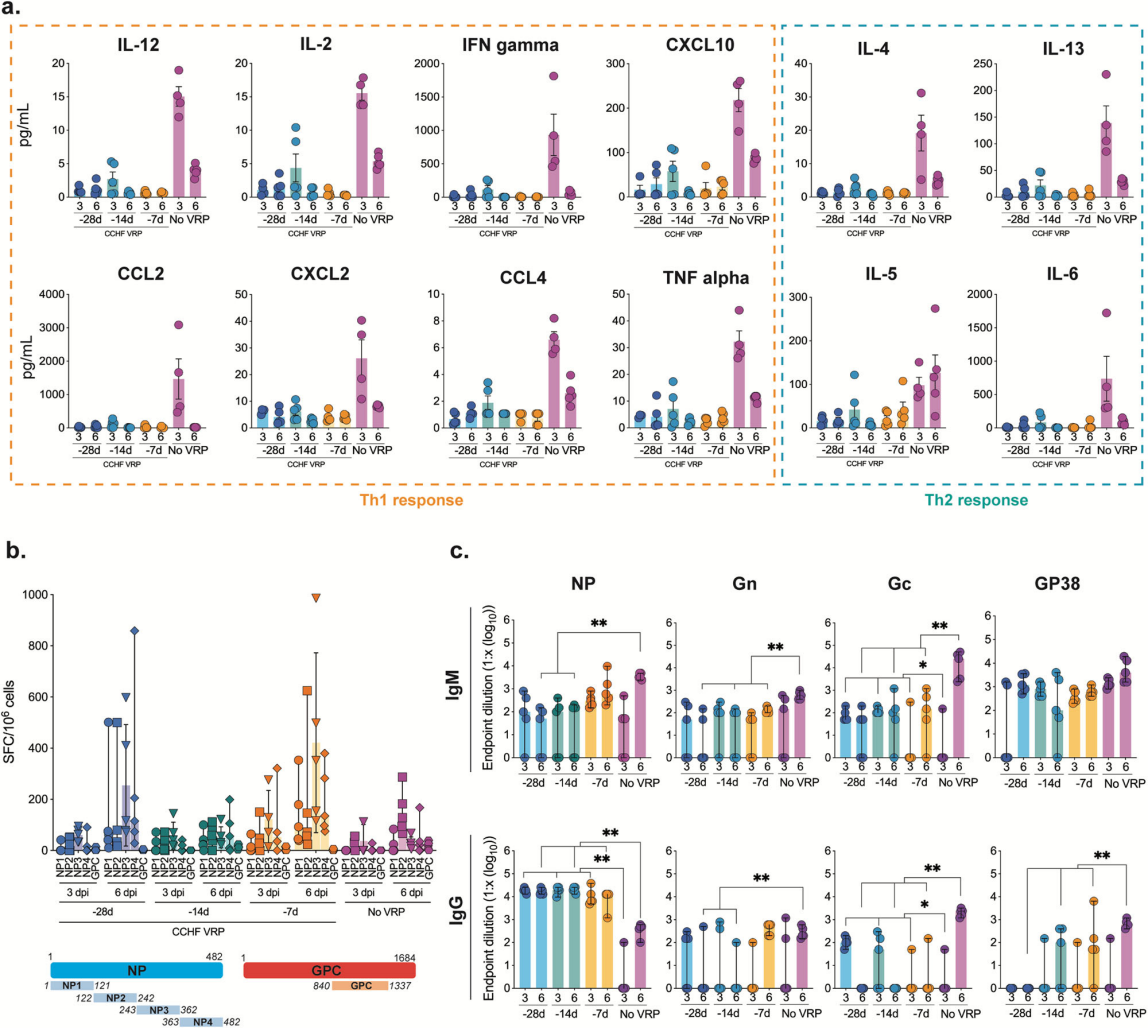
Early control of virus replication is associated with presence of anti-NP antibodies rather than innate or cellular immune responses. **a** Cytokine/chemokine responses in mice vaccinated with CCHF VRP 28, 14, or 7 days prior to challenge or left unvaccinated (no VRP, given DMEM alone) and euthanized 3 or 6 days post infection (dpi) were analyzed using the ProcartaPlex Mouse Th1/Th2 Cytokine and Chemokine panel and 25 µL mouse plasma. **b** CCHFV-specific T-cell responses were evaluated using IFN-gamma ELISpot assay. Peptides covering the CCHFV IbAr10200 NP or Oman-98 GPC NSm-Gc domain (numbers represent aa positions) were used to stimulate splenocytes harvested from vaccinated animals. Data are reported as the number of spot-forming cells (SPC)/1 × 10^6^ cells. **c** CCHFV-specific NP, Gn, Gc, and GP38 antibody responses (IgM and IgG) were evaluated via ELISA using mouse plasma and reported as endpoint dilution titers. Antibody titers, T-cell responses, and cytokine/chemokine levels were compared statistically between vaccine groups by timepoint (3 or 6 dpi) using multiple two-tailed *t*-tests (Mann-Whitney) and only statistically significant results are reported; *p < 0.5, **p < 0.01. Individual animals are represented. Bars and error bars indicate mean ± SEM.

In animals vaccinated with CCHF VRP 28, 14, or 7 days prior to challenge, we found no significant upregulation of cytokines or chemokines at 3 or 6 dpi, corresponding with low levels of viral replication after challenge in these animals (Fig. 7a). Conversely, in non-vaccinated animals, viral replication was widespread as early as 3 dpi, leading to inflammation and elevated cytokines/chemokines (Fig. 7a). NP- and GPC-specific T-cell activation was also low 3 dpi in all groups but increased significantly by 6 dpi in some vaccinated animals (Fig. 7b). Interestingly, T-cell activation was not higher in vaccinated animals than in non-vaccinated controls 3 dpi when significant control of virus replication was evident (Fig. 7b).

In the absence of significant T-cell or innate immune activation 3 dpi, we found robust and significantly higher anti-NP IgG responses in -28D, -14D, and -7D CCHF VRP vaccine cohorts than in control non-survivors; these vaccine groups had similar endpoint dilution titers (1.7 × 10^4^ average) that remained the same from 3 to 6 dpi (Fig. 7c). Comparable titers of anti-NP IgG antibodies developed in LASV VRP vaccinees by day 6 but were not sufficient for protection (Fig. 6c). DMEM-vaccinated controls had widespread CCHFV replication 6 dpi coupled with high-titer anti-CCHFV IgM antibodies, an expected response from animals exposed to CCHFV antigen for the first time. Conversely, we see lower IgM titers in vaccinated animals after challenge corresponding with significantly lower levels of viral replication.

Overall antibody responses to GPC-derived proteins were significantly lower than those generated against NP. At 3 dpi, titers of anti-Gc IgM and IgG antibodies in -28D and -14D vaccine cohorts were significantly higher than in control animals, but endpoint dilution titers were markedly lower than those reported for NP (1.0 × 10^2^) (Fig. 7c). Neutralizing antibody titers were low or undetectable in most animals (Supplementary Fig. 1). Anti-Gn, and anti-GP38 antibodies were not significantly different between vaccine (-28D, -14D, -7D) and control groups until later in infection (6 dpi) (Fig. 7c). Together these data suggest an important role for antibodies, specifically those targeting NP, in long-course CCHF VRP-mediated protective efficacy.

### Anti-NP antibody quality and complement-mediated effector function are highest in animals vaccinated 28 days prior to challenge

**Fig. 8:**
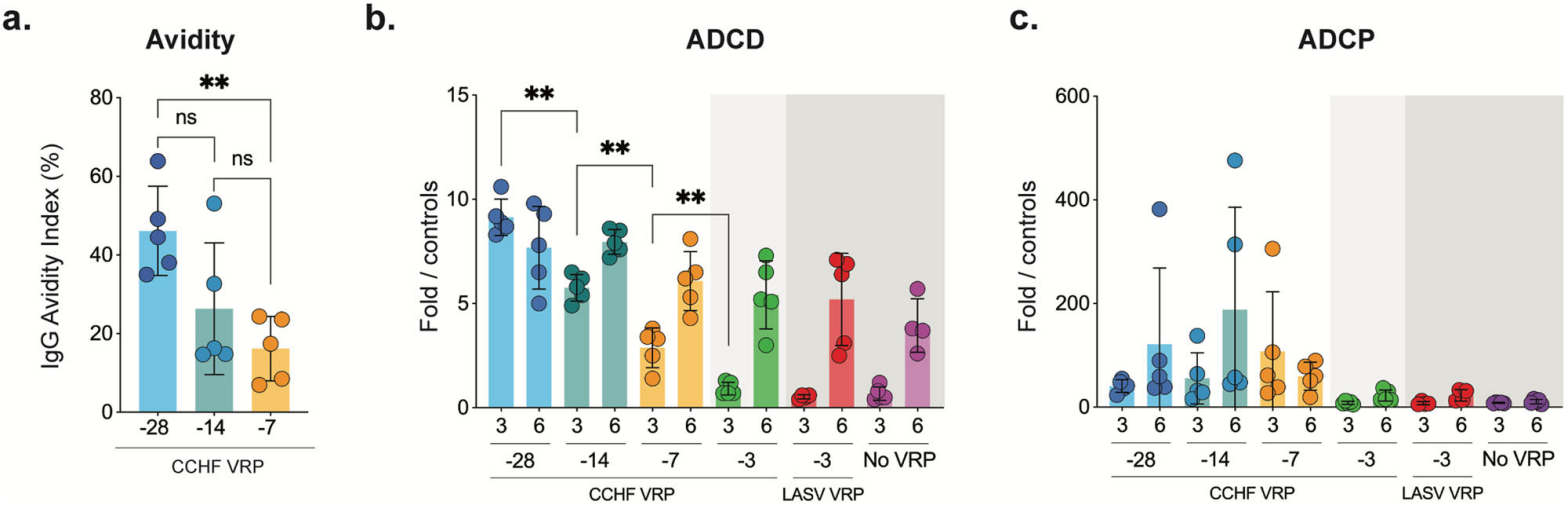
Anti-NP antibody quality and complement-mediated effector function are highest in animals vaccinated 28 days prior to challenge. **a** Anti-NP antibody quality was assessed using an avidity ELISA in which mouse plasma was exposed to varying concentrations of a chaotropic agent (ammonium thiocyanate). Antibody quality was evaluated in plasma from mice vaccinated 28, 14, or 7 days prior to challenge and euthanized 3 dpi. **b** Antibody-dependent complement deposition (ADCD) assay and (**c**) antibody-dependent cellular phagocytosis (ADCP) assays were performed using plasma from mice in all vaccine cohorts at 3 and 6 dpi. Fold ADCD activation was calculated using naïve mouse plasma. ADCP phagocytic score was calculated by multiplying the percentage of bead-positive cells by the overall median fluorescence intensity. Data were compared statistically between vaccine groups by timepoint (3 or 6 dpi) using multiple two-tailed *t*-tests (Mann-Whitney) and only statistically significant results are reported; *p < 0.5, **p < 0.01. Individual animals are represented. Bars and error bars indicate the mean ± standard deviation (SD).

We have shown evidence supporting the role of anti-NP antibodies in CCHF VRP vaccine-mediated protection. While anti-NP antibody titers were all comparable between longer course vaccine groups (-28D, -14D, and -7D) at 3 and 6 dpi, we show that virus replication was most effectively controlled when the vaccine was administered earliest from the time of challenge (-28D). This suggests that if these antibodies contribute to protection, factors other than titer alone are likely important for virus control and clearance. We wondered if differences in antibody quality (avidity), which needs time to develop via affinity maturation, could be detected between vaccine groups. We found that, in fact, anti-NP antibody avidity was highest in animals vaccinated 28 days prior to challenge, and the overall binding strength of anti-NP antibodies was lower in animals vaccinated closer to the time of challenge (Fig. 8a).

The predominance of anti-NP antibodies raises questions regarding the possible mechanisms behind non-neutralizing NP-mediated protection. A growing number of recent publications have also demonstrated the role of Fc-mediated effector function in vaccine efficacy for a wide array of pathogens, including SARS-CoV-2, *Plasmodium* spp., and Ebola virus^40–42^. In the case of CCHFV, studies have shown that non-neutralizing antibodies directed against the GP38 protein can confer protection from lethal disease and that this efficacy depends on the presence of complement^43^. We therefore examined differences in Fc-mediated function of the anti-NP antibodies between long-course vaccine groups. We conducted antibody-dependent complement deposition (ADCD) assays to assess Fc-mediated complement induction, and antibody-dependent cell-mediated phagocytosis (ADCP) to assess Fc-mediated phagocytosis induction (Fig. 8b and c). We found that, indeed, antibody function, specifically ADCD, incrementally increased in groups vaccinated further from the time of infection (Fig. 8b). This was not the case for ADCP responses. In fact, ADCP responses were not statistically different between vaccine and control groups at either timepoint post-challenge (Fig. 8c).

## Discussion

Here, we built upon previous CCHF VRP vaccine efficacy studies by conducting a series of serial euthanasia experiments to investigate how the timing of CCHF VRP vaccination affects viral replication and dissemination, tissue pathology, clinical analytes, and immune responses after CCHFV challenge. Importantly, we identified both platform- and host-specific features associated with CCHF VRP-mediated vaccine efficacy in the Ifnar^-/-^ mouse model of lethal disease.

Rapid protection after vaccination has been reported for other vaccines, including an Ebola virus vaccine that confers protection through non-specific innate immune signaling^31,32^. In vitro studies of CCHFV have also demonstrated the importance of innate signaling, specifically the upregulation of interferon-stimulated genes like MxA and 2′-5′-OAS in inhibiting viral replication^44^. While these data suggest a possible role for non-specific innate immunity in the rapid protection (-3D vaccine group) conferred by the CCHF VRP, our investigations determined that the vaccine platform requires antigen specificity to confer short-course protection.

We further determined that the VRP platform requires single-round replication/transcription for efficacy. Non-replicating virus-like particles (VLP) and inactivated whole virion vaccines have been evaluated in vivo against lethal CCHFV infection with varying degrees of success, conferring 40–100% protection^45,46^. Our data suggest that, as reported for a similar Lassa VRP vaccine^29,33^, CCHF VRP vaccine efficacy requires additional antigen production and/or cell signaling induced by replication/transcription events to generate protective immunity.

Serial sampling data demonstrated that reduction or elimination of clinical disease after infection in CCHF VRP vaccinated animals was associated with reduced viral load and corresponding preserved liver function. Vaccination as late as 3 days before infection reduces CCHFV replication in tissues and mucosal swabs compared to control animals. Importantly, vaccination 7 or more days prior to challenge eliminates viral antigen detection outside of liver and lymphoid tissues, indicating protection from disseminated infection. The liver is a major target organ for CCHFV replication, and the extent of liver damage is known to correspond with negative survival outcomes in humans. Indeed, liver enzyme values are recommended for use as prognostic indicators and triage of CCHF patients in a clinical setting^47,48^. Likewise, CCHF viral load is also often associated with disease severity and negative outcomes in human cases^49–51^. Both viral load and degree of liver damage are critical contributors to CCHF disease severity and outcome, and we show that in all vaccine groups, including short-course VRP-vaccinated animals with mild clinical disease, these parameters are significantly controlled.

To our knowledge, this is the first report to evaluate virus shedding via mucosal swabs in a mouse model of CCHF disease. Previous reports from humans and NHPs have shown that CCHFV RNA can be detected in both oral and rectal swab samples via RT-qPCR; however, data correlating these results to levels of infectious virus are lacking^35^. We recovered infectious virus from rectal (3 of 5 control animals, 6 dpi) but not oral swabs and showed that virus isolation corresponded with higher levels of detectable vRNA. While human-to-human transmission of CCHFV is thought to be infrequent^52,53^, it does occur in nosocomial settings, and these case clusters are associated with high case fatality rates^9,12,36–38^. More data are needed to fully appreciate the role of mucosal shedding in human CCHFV transmission, but small animal models can be advantageous for studying this in more detail. Reducing infectious virus at mucosal surfaces in animals vaccinated with CCHF VRP is potentially significant, given the frequency and lethality of nosocomial outbreaks and transmission.

While we find our investigation into immune correlates of protection to be important, one of the limitations is our use of immunodeficient Ifnar^-/-^ mice. In general terms, data indicate that a lack of Ifnar signaling does not change T-cell numbers or percentages (CD4^+^,CD8^+^, and Treg T-cells) in the liver and spleen compared to C57BL/6 J mice^54^. Conversely, Ifnar^-/-^ mice develop increased numbers of plasma cells and antibodies that have broader reactivity compared to immunocompetent mice^55^. Despite this knowledge, it is not possible to predict specific differences in T- or B-cell responses after vaccination based on the mouse model. While the Ifnar^-/-^ model was necessary to build upon our previous work, most other animal models of lethal CCHF also require the suppression or removal of type I IFN responses and these models do reliably recapitulate critical features of fatal human CCHFV infection, including a distinctive pattern of immune dysregulation featuring elevated levels of IL-6, TNF-alpha, IL-18, and CCL2^17–20^.

We were not able to show differences in innate or T-cell-mediated immune activation between short-course (-3D) CCHF VRP and LASV VRP vaccine groups after challenge, but higher total anti-NP antibody titers (IgM and IgG) were detected in the CCHF VRP group by 3 dpi, indicating that these responses may play a role in rapid vaccine-mediated protection. The appearance of IgG antibodies 3 dpi (6 d after primary antigen exposure) was rapid and surprising. CCHFV challenge serves as a secondary antigen exposure or boost and anamnestic responses induced by secondary immune activation have been shown to result in a rapid (<7 days) increase of IgG antibody titers in the context of other vaccines^56,57^ We speculate that anamnestic responses could explain the rapid appearance of IgG antibodies in the -3D VRP vaccine group, but further investigation would be needed to confirm this hypothesis.

Of the longer-course (-28D, -14D, -7D) vaccine groups, we found that virus replication and dissemination were most effectively controlled in animals vaccinated 28 days prior to challenge, indicating that vaccine-mediated immune responses that require maturation or development over time are most advantageous for preventing CCHF. The quality of antibody binding, or binding strength, which requires development over time though the process of affinity maturation, has been shown to correlate with vaccine efficacy in numerous studies^58,59^. Indeed, high-avidity (high-quality) anti-NP antibodies were most associated with enhanced efficacy in our study.

A lack of neutralizing responses and predominance of anti-NP antibodies demonstrates that non-neutralizing mechanisms have a role in CCHF VRP-mediated protection from lethal CCHF. This is not surprising, as many studies in both humans and mice suggest that neutralizing antibodies do not correlate with protection from CCHF^60^. Anti-NP antibodies have been shown to confer vaccine-mediated protection against other viruses, like influenza and lymphocytic choriomeningitis virus^61,62^; however, the mechanisms behind anti-NP mediated protection need elucidation. In the case of CCHFV, protective non-neutralizing antibodies targeting the GP38 protein have also been described^27,43,63^ and shown to depend on the presence of complement for efficacy^43^. Our data also suggest a role for complement (ADCD) in antibody-mediated efficacy of CCHF VRP vaccination.

Interestingly, studies have shown a clear and strong relationship between high levels of antibody avidity and Fc-mediated complement activity^64^. High avidity is required to achieve optimal immune complex stoichiometry in which Fc-Fc interactions occur and assembly into ordered antigen-bound hexamers creates the optimal structure to bind and activate complement^65^. Conversely, antibodies with low or moderate avidity predominantly bind monovalently and thus tend to elicit stronger effector functions like ADCP because they result in higher cell surface densities of unassociated Fc domains^64^. These unassociated Fc domains resulting from low-avidity monomeric binding can only weakly associate with complement protein C1 and result in highly transient interactions. Thus, higher avidity is associated with higher levels of ADCD activity. These data are complementary to our findings that, as antibody avidity increases, ADCD activity increases while ADCP activity does not. These findings warrant further investigation into the function of complement during CCHFV infection.

Given our use of Ifnar^-/-^ mice, a useful point of comparison for reviewing our correlates of protection data comes from a recent study investigating immune responses in immunocompetent C57BL/6 mice after CCHF VRP vaccination alone^66^. Consistent with our findings, Scholte et al. showed a predominance of anti-NP antibodies with detectable ADCD effector function and minimal GPC targeted antibodies. Scholte et al. also found that T-cell responses (IFN-gamma) were most significant against NP and peaked 5–7 days post vaccination; the magnitude of these responses was higher overall than those reported here (in Ifnar^-/-^ mice) after vaccination and challenge. Due to this difference, we cannot discount the potential role for T-cell responses in the efficacy of the VRP in immunocompetent recipients. However, our study clearly demonstrates that anti-NP humoral responses are sufficient to confer total protective efficacy in the absence of significant innate or T-cell responses, raising questions about the necessity of T cell responses for efficacy. We would caution that our investigations into T-cell responses are limited to IFN-gamma production alone, which may overlook other responses that could play a role in protection, like TNF-alpha or IL-2. Further investigations into the role of T-cells in CCHF VRP efficacy are warranted.

Critically, in this study, we identified several important components of vaccine-mediated protection, including the presence of high-quality anti-NP antibodies capable of mediating ADCD. Future studies, including use of knockout murine strains, would be useful to provide further support for our findings and determine additional correlates of CCHF VRP vaccine-mediated protection. These data provide a narrowed scope and direction for future studies on the correlates of protection of the CCHF VRP vaccine platform and for defining benchmarks of success for future CCHF vaccine development efforts.

## Methods

### Biosafety and ethics statement

All experiments involving CCHFV were conducted in the biosafety level 4 (BSL-4) laboratory at the Centers for Disease Control and Prevention (CDC; Atlanta, GA, USA). Experiments involving cDNA-encoding viral sequences were performed in accordance with approved Institutional Biosafety Committee protocols. Animal studies were conducted in compliance with the *Guide for the Care and Use of Laboratory Animals* and approved by the CDC Institutional Animal Care and Use Committee (IACUC; #3034). The CDC is fully accredited by AAALAC International.

### VRP vaccine constructs

CCHF and LASV VRP stocks were generated as previously described^29,33^. Briefly, CCHF VRP were generated by transfecting HuH-7 cells with plasmids encoding CCHFV strain IbAr10200 S and L genomic segments (pT7-S and pT7-L) and strain Oman-98 glycoprotein (pCAGGS-GPC-Oman) along with plasmids encoding the polymerase (codon optimized pCAGGS-L), nucleoprotein (pCAGGS-NP), and T7 polymerase (pCAGGS-T7). Supernatants were harvested and VRP were quantified via immunofluorescent TCID_50_ assay using BSRT7/5 cells (provided by K.K. Conzelmann, Ludwig-Maximilians-Universität, Munich, Germany); immunostaining was performed using a rabbit polyclonal anti-NP CCHFV antibody (1:2500; IBT, 04-0011) followed by a goat anti-rabbit secondary antibody (1:2500; Alexa Flour 488, Invitrogen, A-11008). Wells were scored visually for the presence or absence of fluorescent cells and TCID_50_ values were calculated using the Reed-Muench method. When indicated, CCHF VRP stocks were UV-inactivated using the Spectronics Corp Spectrolinker UV Crosslinker with a total exposure dose of 800 mJ/cm^2^ (2 × 400 mJ/cm^2^). UV inactivation is a well-described method for denaturing viral genomic material while preserving features required for particle entry (including membrane and protein structure), ensuring that the virus can enter the cells but not undergo further rounds of replication^67,68^. All vaccine stocks were verified to be mycoplasma free (MycoAlert PLUS detection kit, Lonza LT07) and genomic sequences were confirmed via NGS prior to in vivo studies.

### Challenge virus

CCHFV strain Turkey04 (Turkey-200406546; GenBank accession nos. KY362517, KY362519, KY362515; Stock #813732) was isolated from a hospitalized human patient whose clinical outcome is unknown. The virus was previously passaged once in suckling mouse brain and once in SW-13 cells. Stock and inoculum titers were calculated via immunofluorescent TCID_50_ (Reed Muench method) assay using BSR-T7/5 cells (described above).

### In vivo experiments

The mouse strain used for this research project, B6.129S2-Ifnar1tm1Agt/Mmjax, RRID:MMRRC_032045-JAX, was obtained from the Mutant Mouse Resource and Research Center (MMRRC) at The Jackson Laboratory, an NIH-funded strain repository, and was donated to the MMRRC by Michel Aguet, Ph.D., Swiss Institute for Experimental Cancer Research. Groups of 8–10 mice (mixed sex, 4–7 weeks of age) were vaccinated SC with CCHF VRP, UV-inactivated CCHF VRP, or LASV VRP (1.00 × 10^5^ TCID_50_), or remained unvaccinated (no VRP, given DMEM alone) 28, 14, 7, or 3 days prior to SC challenge with lethal CCHFV Turkey04 (target dose: 100 TCID_50_; actual dose: 55 TCID_50_). Cohorts from each group (except UV-CCHF VRP, *n* = 4−5) were serially euthanized 3 or 6 dpi. Groups of LASV VRP- and UV-inactivated CCHF VRP-vaccinated animals (*n* = 5 each) were also followed to determine survival outcome.

Mice were housed in a climate-controlled laboratory with a 12 h day/night cycle; provided sterilized commercially available mouse chow and sterile water *ad libitum*; and group-housed on autoclaved corn cob bedding (Bed-o’Cobs ¼”, Anderson Lab Bedding) with cotton nestlets in an isolator-caging system (Tecniplast GM500 cages) with a HEPA-filtered inlet and exhaust air supply. Mice were evaluated daily for clinical signs of disease and assigned a score ranging 0–10 based on the following criteria: piloerection, hunched posture, hypoactivity, percent weight loss, abnormal respiration, dehydration, and neurological signs (ataxia, paresis). Euthanasia criteria were met when weight loss exceeded 25% from baseline (1 day prior to infection) and/or the clinical score reached 10. Mice were humanely euthanized via isoflurane exposure followed by cervical dislocation at the serial timepoints indicated or when meeting euthanasia criteria according to protocols approved by CDC’s IACUC.

### Clinical chemistry

Non-fasting whole blood samples from each animal were collected peri-mortem via intracardiac bleed, placed in lithium heparin (LiH), and immediately analyzed via the General Chemistry 13 Panel on the Piccolo Xpress analyzer (Abaxis).

### RT-qPCR

RNA was extracted from LiH whole blood (50 µL), mucosal swabs, or homogenized tissue (liver, spleen, gonad, kidney, heart, lung, eye, brain) in MagMAX lysis buffer. MagMAX Pathogen RNA/DNA kits (Thermo Fisher, 4462359) were used in conjunction with the 96-well ABI MagMAX extraction platform; RNA was eluted into 75 µL of elution buffer. Viral RNA was quantified using a primer/probe set targeting the CCHFV Turkey04 strain NP ORF of the S genomic segment (forward: CAACAGGCTGCTCTCAAGTG; reverse: CAATTTCGCCAGGGACTTTA; probe: 56FAM/ACACGGCAG/ZEN/CCTTAAGCAACAA/3IABkFQ; IDT) using the SuperScript III Platinum One-Step RT-qPCR kit (Thermo Fisher). An average 18S Ct value was calculated for each tissue type (Thermo Fisher) and used to normalize the Ct values from each sample. Viral RNA copy numbers were then quantified via standard curve generated from an RNA standard of known concentration (IDT). Data are reported as S genome copy number/µL RNA.

### Virus isolation and quantification

Oropharyngeal and rectal swabs (Puritan Sterile Miniature Polyester Tipped Applicator; Puritan Medical Products, 25-800 1PD 50) collected for virus isolation were placed in serum-free DMEM supplemented with 2 × antimycotic/antibiotic (Gibco) and allowed to sit at room temperature (RT) for 15–20 min. Samples were centrifuged to clear particulates, then plated (100 µL/well) in triplicate onto BSR-T7/5 cells seeded into 12-well plates. Plates were incubated at 37°C and rocked every 15 min for 1 h. DMEM (1 mL) supplemented with 5% FBS and 2 × antibiotic/antimycotic was added to each well before plates were incubated at 37°C for 5 days. Plates were fixed with 4% formaldehyde and permeabilized with 0.1% Triton-X-100, followed by immunostaining with a polyclonal rabbit anti-NP CCHF antibody (1:2500; IBT, 04-0011) and goat anti-rabbit secondary antibody (Alexa Flour 488; Invitrogen, A-11008). Samples were determined to be positive for CCHFV if one or more fluorescent foci were visually present. Positive samples were further analyzed via TCID_50_ assay (described above) to quantify infectious virus. The limit of detection for virus isolation in this assay was 21.7 TCID_50_/mL.

### Cytokine/Chemokine analyses

Plasma samples from each animal were gamma-irradiated (5.0 × 10^6 ^rad dose) and analyzed (25 µL) using the ProcartaPlex Mouse Th1/Th2 Cytokine and Chemokine 20-plex panel (Thermo Fisher, EPX200-26090-901). Data were read using the Luminex 200 analyzer. Trends in data were consistent across analytes but only a subset (*n* = 12) are shown because these are associated with CCHFV infection in vivo^60^.

### Protein expression and ELISA

CCHFV strain Kosovo Hoti sequences were utilized for protein expression (GenBank accession nos. DQ133507.1 [S segment], EU037902.1 [M segment]). Several publications have demonstrated high levels of CCHFV NP cross-reactivity across clades due to its high sequence conservation; thus, we are confident in the performance of the NP ELISA using a heterologous virus strain^69–71^. While CCHFV GPC is more variable across clades, strain Hoti has 95.32% amino acid sequence identity and 97% amino acid sequence similarity to the M segment (GPC) of the challenge virus strain (Turkey04). We have also demonstrated that GP38 antibodies from CCHFV Turkey04-infected animals result in similar ELISA endpoint titers using GP38 protein from strain Hoti and Turkey04, confirming a high level of cross reactivity (Supplementary Fig. 2).

The sequence for expressing CCHFV nucleoprotein was optimized for bacterial expression and cloned into pET28a by Twist Bioscience. The construct was transformed in *Escherichia coli* strain BL21 (DE3) (Thermo Fisher) and bacterial culture was grown in Luria broth with kanamycin. The culture was induced with 1 mM isopropyl β-D-1-thiogalactopyranoside when optical density was between 0.4–0.6. Following induction, the culture was transferred to 16°C for overnight incubation. Cells were harvested with centrifugation, resuspended in lysis buffer (500 mM NaCl, 20 mM Tris-Cl [pH 7], 0.1% Triton-X, 5% glycerol, 1 mM MgCl_2_, 25 U/mL benzonase), and sonicated. CCHFV Gn, Gc, and GP38 sequences were cloned into pTWIST (Gn) or pEEV (Gc and GP38) plasmids by Twist Bioscience. Proteins were expressed in Expi293F cells (Thermo Fisher) grown in Expi293 Expression medium with transient transfection using FectoPro transfection reagent according to the manufacturer’s recommendations (Polypus). Briefly, cells were transfected with 0.8 μg plasmid/mL of culture using 1.5 μL of transfection reagent/μg of the plasmid. After 4–6 days, cells were harvested and culture supernatants were processed for protein purification. The cleared bacterial lysates and mammalian cell culture supernatants were filtered through a 0.2 micron polyethersulfone membrane and loaded onto the HisTrap Excel column (Cytiva) for immobilized metal affinity chromatography. Proteins were further purified with size exclusion chromatography (Superdex 200 increase 16/600 GL, Cytiva), quantified, and stored at -80°C.

For ELISA, Immulon 2HB plates were coated with 100 μL of 500 ng/mL antigen prepared in phosphate buffered saline (PBS) and incubated overnight at 4°C. Wells were washed 4 × with 300 μL PBST (0.1% Tween-20 in PBS) and blocked (5% [w/v] non-fat dry milk in PBST) for 1 h at RT. Following blocking, buffer was decanted and 100 μL of mouse plasma prepared in blocking buffer with 2-fold serial dilutions (range 1:200 to 1:1024000) was added to the wells in duplicate. After 1 h incubation at RT, wells were washed 4 ×, anti-mouse IgG HRP (1:3000) or anti-mouse IgM HRP (1:1000) was added to the wells (100 μL), and plates were incubated for 1 h at RT. Following incubation, wells were washed 4 × and 100 μL TMB Ultra ELISA substrate (Thermo Fisher) was added and incubated for 10 min at RT. The reaction was stopped by adding ELISA stop solution (Thermo Fisher), and optical density was read at 450 nm on a Synergy Neo2 instrument (BioTek) microplate reader. A cut-off value was determined for each plate based on the average absorbance value of negative control wells plus 3 standard deviations. The highest dilutions with a signal above the determined cut-off value were assigned as the endpoint titers.

### Neutralizing antibodies

Two-fold dilutions (1:10 to 1:1280) of heat-inactivated (56°C, 30 min) mouse plasma were incubated (1:1) for 1 h with 200 TCID_50_ of CCHFV Turkey04 or the CCHF VRP vaccine construct encoding a ZsGreen (ZsG) reporter and then transferred onto BSRT7/5 cells for 5 and 3 days, respectively, at 37 °C. Virus presence was determined by fluorescent signal from ZsG expression (CCHF VRP) or immunostaining (CCHFV Turkey04; see “Virus Isolation and Quantification” section). Neutralizing titers were performed in technical quadruplicate and recorded as the lowest reciprocal dilution in which all 4 wells were free of fluorescent signal. Titers were normalized to background neutralization from normal age-matched mouse plasma. Each reaction was performed in technical quadruplicate.

### Antibody avidity

Avidity ELISAs were performed as described above with an additional treatment step using a chaotropic agent. All samples were prepared in 4 replicates; after incubating serum samples, the first replicate was treated with 1 M ammonium thiocyanate (NH_4_SCN), and the second was treated with PBS for 10 min at RT. The plates were washed, and the assay was completed as described above. The avidity index was calculated by dividing the OD of the NH_4_SCN-treated sample by that of the PBS-treated one and multiplied by 100.

### Antibody function

ADCD assays were adapted from Fishinger et al. (2019)^72^. Recombinant CCHFV NP (Hoti strain, GenBank accession no. DQ133507.1) was biotinylated (21435, EZ-Link™ Sulfo-NHS-LC-Biotinylation Kit) and coupled to 1.0 µm fluorescent red neutravidin microspheres (Thermo Fisher F8775). Excess antigen was washed away with PBS containing 5% BSA. Antigen-coated beads were incubated with mouse plasma (2 h at 37°C) and unbound antibodies were washed away with PBS. Guinea pig complement (Cedarlane, CL4051) diluted in gelatin veronal buffer (CompTech B102) was added and incubated 15 min at 37°C. Immune complexes were washed with 15 mM EDTA in PBS and incubated 15 min at RT with FITC-conjugated goat IgG fraction to guinea pig complement C3 (MP Biomedicals, 0855385). Unbound antibodies were washed away with PBS and immune complexes were analyzed on a Guava Cytometer. Fold ADCD activation was calculated using naïve mouse plasma.

ADCP assays were adapted from Butler et al. (2019)^73^. Immune complexes were formed as described for ADCD, except biotinylated antigen was coupled to 1.0 µm fluorescent green neutravidin microspheres (Thermo Fisher F8776). Excess antigen was washed away with PBS containing 5% BSA. Antigen-coated beads were incubated with mouse plasma (2 h at 37°C) and unbound antibodies were washed away with PBS. Immune complexes were incubated overnight at 37°C with 1 × 10^4^ THP1 cells per well. The next day, cells were washed and analyzed on a Guava Cytometer. Phagocytic score was calculated by multiplying the percentage of bead-positive cells with the overall median fluorescence intensity and dividing that number by 10,000.

### Histology and immunohistochemistry

Tissue specimens were fixed in 10% neutral buffered formalin and gamma-irradiated (5 × 10^6 ^rad). Tissues were routinely processed for paraffin embedding, sectioning, and staining with hematoxylin and eosin. Immunohistochemical assays were performed using indirect immunoalkaline phosphatase detection. Briefly, 4 μm tissue sections were placed on slides, deparaffinized in xylene, and rehydrated through graded alcohol solutions. Colorimetric detection was performed using the Mach 4 AP Polymer kit (Biocare Medical, Pacheco, CA, USA). All steps of the staining procedure were performed at RT. Slides were digested with 0.1 mg/mL proteinase K in 0.6 M tris/0.1% CaCl_2_ for 15 min. All slides were then blocked in Background Punisher (Biocare Medical, Pacheco, CA) for 10 min and incubated with a rabbit anti-CCHFV N pAb (IBT Bioservices, Rockville, MD; #04-0011) diluted 1:1000 for 30 min. Mach 4 AP polymer was applied for 30 min. The antibody/polymer conjugate was visualized by applying Sigmafast Fast Red Chromogen (Millipore Sigma) to tissue sections for 30 min. Slides were counterstained in Mayer’s Hematoxylin (PolyScientific) and stained blue with lithium carbonate (Polysciences). Slides were cover-slipped using aqueous mounting medium (Polysciences, Inc.).

Liver inflammation and necrosis and splenic lymphoid reactivity were scored semi-quantitatively as follows. Liver inflammation: 0 = none; 1 ≤ 3 small foci; 2 ≤ 5 medium sized discrete foci or few widespread cells; 3 ≥ 5 discrete foci or widespread moderate; 4 = numerous discrete foci or widespread severe. Liver necrosis: 0 = none; 1 ≤ 3 small foci, single-cell or confluent; 2 ≥ 3 foci, single-cell or confluent; 3 = widespread moderate, single-cell or larger confluent foci; 4 = extensive single-cell and large confluent foci. Splenic lymphoid reactivity: 0 = none; 1 = minimal; 2 = mild; 3 = moderate; 4 = severe. The scorer was blinded to treatment groups.

### IFN-gamma ELISpot assay

Spleens were isolated from euthanized animals and processed into a single-cell suspension using the GentleMACS tissue dissociator. Red blood cells were lysed using RBC lysis buffer. Cell suspensions were frozen, thawed in RPMI, diluted to 2 × 10^6^ cells/mL, and seeded in MabTech ELISpot plates containing peptides homologous to the VRP vaccine construct, covering the CCHFV IbAr10200 NP (AbClonal; 15-mers with 11 AA overlap) or Oman-98 GPC NSm-Gc domain (AbClonal; 15-mers with 4 AA overlap). PMA was used as a positive control and DMSO as a negative control. Splenocytes were incubated for 48 h before spot development according to the manufacturer’s instructions. Spots from each animal were counted using a CTL ELISpot reader, background reactivity subtracted, and normalized to PMA-induced positive control wells.

### Statistical analyses

All statistical analyses were conducted using GraphPad Prism version 9.1.2.

### Reporting summary

Further information on research design is available in the Nature Research Reporting Summary linked to this article.

### Supplementary information

Supplementary Materials

Reporting Summary

## Data Availability

All data are available in the manuscript or the Supplementary materials. Correspondence and requests for materials should be addressed to JRS (wsk7@cdc.gov).
