# Supplementary material for: Replicon particle vaccination induces non-neutralizing anti-nucleoprotein antibody-mediated control of Crimean-Congo hemorrhagic fever virus
Source: NPJ Vaccines. 2024 May 23;9:88. doi: 10.1038/s41541-024-00877-1 (PMC11116556; doi:10.1038/s41541-024-00877-1)
Supplement: Supplementary file 1 — Supplementary Materials [file 41541_2024_877_MOESM1_ESM.pdf]

Replicon particle vaccination induces non-neutralizing anti-nucleoprotein antibody-mediated control of Crimean-Congo hemorrhagic fever virus

Teresa E. Sorvillo[1,2], Elif Karaaslan[1,3], Florine E. M. Scholte[1], Stephen R. Welch[1], JoAnn D. Coleman-McCray[1], Sarah C. Genzer[1], Jana M. Ritter[4], Heather M. Hayes[4], Shilpi Jain[1], Scott D. Pegan[3], Éric Bergeron[1], Joel M. Montgomery[1], Christina F. Spiropoulou[1], Jessica R. Spengler[1*]

Affiliations:
[1]Viral Special Pathogens Branch, Division of High Consequence Pathogens and Pathology, Centers for Disease Control and Prevention, 1600 Clifton Road, Atlanta, GA, USA
[2]Infectious Disease Department, CDC Foundation, Atlanta, GA, USA
[3]Division of Biomedical Sciences, University of California Riverside, Riverside, CA, USA
[4]Infectious Diseases Pathology Branch, Division of High Consequence Pathogens and Pathology, Centers for Disease Control and Prevention, 1600 Clifton Road, Atlanta, GA, USA

Supplementary Figures/Tables

Supplementary Figure 1. Neutralizing antibodies are low or undetectable in mice after CCHF VRP vaccination alone and in vaccinated mice following virus challenge.

Supplementary Figure 2. Strain Turkey04 anti-GP38 antibodies are detected at comparable levels using GP38 proteins from CCHFV strain Turkey04 or Kosovo Hoti.

Supplementary Table 1. Statistical comparisons (p-values) of tissue and swab viral RNA from CCHF VRP-vaccinated versus unvaccinated mice.

Supplementary Table 2. Statistical comparisons (p-values) of clinical chemistry values from CCHF VRP-vaccinated versus unvaccinated mice.

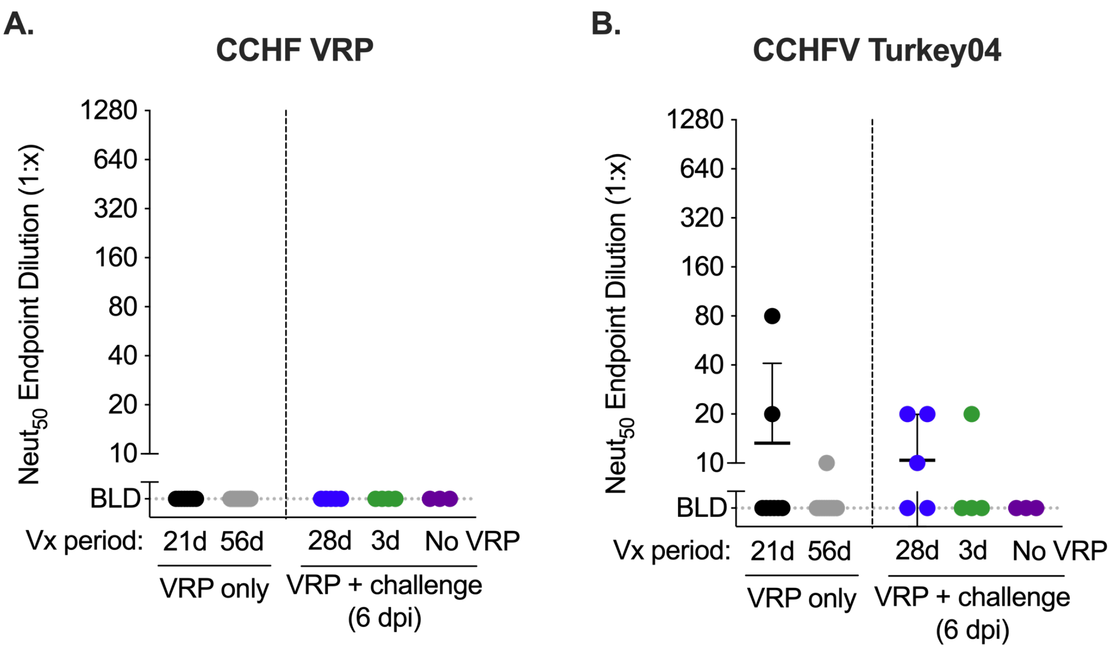

Supplementary Figure 1. Neutralizing antibodies are low or undetectable in mice after CCHF VRP vaccination alone and in vaccinated mice following virus challenge. Neutralizing activity in plasma collected from immunocompetent C57BL/6 mice that received VRP alone was quantified against (A) CCHF VRP vaccine (expressing a ZsGreen reporter) or (B) challenge virus (wild-type CCHFV strain Turkey04 [Turkey-200406546]) at 21 or 56 days post vaccination. Similarly, neutralizing antibody titers were also assessed in Ifnar$^{-/-}$ mice euthanized at 6 days post-infection [(target dose: 100 $TCID_{50}$, subcutaneous inoculation, strain Turkey04 (Turkey-200406546)] that were VRP vaccinated (28 or 3 days prior to challenge) or mock vaccinated (no VRP, DMEM alone). Neutralization was recorded as the lowest reciprocal dilution in which all four replicates were free of fluorescent signal. Bars represent mean and standard deviation of each dataset.

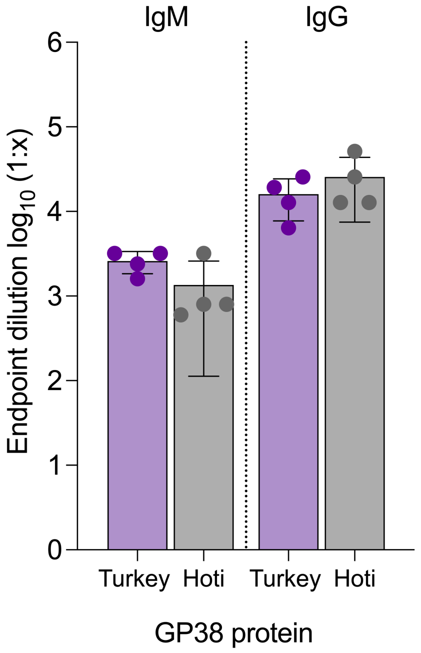

Supplementary Figure 2. Strain Turkey04 anti-GP38 antibodies are detected at comparable levels using GP38 proteins from CCHFV strain Turkey04 or Kosovo Hoti. IgM and IgG GP38 antibody responses from mouse plasma were quantified via ELISA. Plasma samples (n = 4) were obtained from C57BL/6J mice infected with CCHFV strain Turkey04 (Turkey-200406546) at 14 days post-infection. ELISA plates were coated with GP38 protein from either CCHFV strain Kosovo Hoti or Turkey04 to compare cross-reactivity. Data are reported as endpoint dilution titers and compared statistically using multiple t-tests (Mann-Whitney). No statistically significant differences were detected. Bars represent mean and standard deviation of each dataset.

Supplementary Table 1. Statistical comparisons (p-values) of tissue and swab viral RNA from CCHF VRP-vaccinated versus unvaccinated mice

	3 days post-infection					6 days post-infection				
	-28D vs. No VRP	-14D vs. No VRP	-7D vs. No VRP	-3D vs. No VRP	LASV vs. No VRP	-28D vs. No VRP	-14D vs. No VRP	-7D vs. No VRP	-3D vs. No VRP	LASV vs. No VRP
**Liver**	0.0079	0.0079	0.0079	0.0079	0.2857	0.0079	0.0079	0.0079	0.0079	0.5556
**Spleen**	0.0079	0.0079	0.0079	0.0556	0.4127	0.0079	0.0079	0.0079	0.0079	0.1905
**Gonad**	0.0079	0.0079	0.0079	0.2222	0.6111	0.0079	0.0079	0.0079	0.0079	0.4127
**Kidney**	0.0079	0.0317	0.0159	0.8413	0.5556	0.0079	0.0079	0.0079	0.0079	0.5556
**Heart**	0.0079	0.0159	0.0079	0.2222	0.1905	0.0079	0.0079	0.0079	0.0079	0.5556
**Lung**	0.0079	0.0317	0.0238	0.2222	0.4127	0.0079	0.0079	0.0079	0.0079	0.4127
**Eye**	0.0079	0.0873	0.0079	0.2222	0.9048	0.0079	0.0079	0.0079	0.0079	0.2857
**Brain**	0.0079	0.0079	0.0159	0.0556	>0.9999	0.0079	0.0079	0.0079	0.0079	0.1905
**Blood**	0.0079	0.0079	0.0079	0.0476	0.5556	0.0079	0.0079	0.0079	0.0159	0.7302
**Oral sw**	0.1667	0.1667	0.1667	0.2857	>0.9999	0.0079	0.0079	0.0079	0.3095	0.7302
**Rectal sw**	0.0476	0.1032	0.0476	0.6905	0.8492	0.0079	0.0079	0.0079	0.0079	0.0635

Data found in Figures 2C and 3A. Cohorts of Ifnar-/- mice (n = 8–10) were vaccinated SC with CCHF VRP or LASV VRP (both at 1×10^5 $TCID_{50}$), or left unvaccinated (no VRP, given DMEM alone) 28, 14, 7, or 3 days (-28D, -14D, -7D, -3D) prior to challenge with lethal CCHFV Turkey04 (1×10^2 $TCID_{50}$, SC). Tissues and mucosal swabs were collected from all mice at the time of serial euthanasia 3 or 6 dpi. Viral RNA was isolated and quantified via RT-qPCR using primers/probe specific for the NP ORF of the CCHFV S gene segment. Statistics for each vaccine group were calculated as significant change compared to unvaccinated control animals (No VRP, given DMEM alone) at equivalent timepoint (3 or 6 dpi) using multiple t-tests (Mann-Whitney). Red indicates $p < 0.05$.

Supplementary Table 2. Statistical comparisons (p-values) of clinical chemistry values from CCHF VRP-vaccinated versus unvaccinated mice

	3 days post-infection					6 days post-infection				
	**-28D vs. No VRP**	**-14D vs. No VRP**	**-7D vs. No VRP**	**-3D vs. No VRP**	**LASV vs. No VRP**	**-28D vs. No VRP**	**-14D vs. No VRP**	**-7D vs. No VRP**	**-3D vs. No VRP**	**LASV vs. No VRP**
**GLU**	0.0159	0.0317	0.0079	0.3095	0.5714	0.0079	0.0079	0.0079	0.1508	0.9048
**BUN**	0.0159	0.1587	0.3333	0.0397	0.7143	0.0079	0.0159	0.0159	0.4683	0.9127
**ALT**	0.0317	0.0952	0.0317	0.2222	0.5714	0.0952	0.0079	0.0079	0.0159	0.1111
**AST**	0.0556	0.2222	0.0952	0.4206	>0.9999	0.0159	0.0079	0.0079	0.0317	0.2857
**ALB**	0.2778	0.1905	0.0397	0.3413	0.8393	0.0079	0.0079	0.0159	>0.9999	>0.9999
**TP**	0.0238	0.0794	0.0079	0.1270	0.3036	>0.9999	0.4048	0.2540	0.8333	0.5397

Data found in Figure 3B. Cohorts of Ifnar-/- mice (n = 8–10) were vaccinated SC with CCHF VRP or LASV VRP (both at 1×10^5 $TCID_{50}$), or left unvaccinated (no VRP, given DMEM alone) 28, 14, 7, or 3 days (-28D, -14D, -7D, -3D) prior to challenge with lethal CCHFV Turkey04 (1×10^2 $TCID_{50}$, SC). Whole blood was collected in lithium heparin from all mice at the time of serial euthanasia (3 or 6 dpi) and analyzed via General Chemistry 13 Panel on the Piccolo Xpress analyzer. Statistics for each vaccine group were calculated as significant change compared to unvaccinated control animals (No VRP, given DMEM alone) at equivalent timepoint (3 or 6 dpi) using multiple t-tests (Mann-Whitney). Red indicates $p < 0.05$. GLU, glucose; BUN, blood urea nitrogen; ALT, alanine aminotransferase; AST, aspartate aminotransferase; ALB, albumin; TP, total protein.
