# Supplementary material for: Replicon particle vaccination induces non-neutralizing anti-nucleoprotein antibody-mediated control of Crimean-Congo hemorrhagic fever virus
Source: NPJ Vaccines. 2024 May 23;9:88. doi: 10.1038/s41541-024-00877-1 (PMC11116556; doi:10.1038/s41541-024-00877-1)
Supplement: Supplementary file 2 — Reporting Summary [file 41541_2024_877_MOESM2_ESM.pdf]

nature portfolio

Corresponding author(s): Jessica Spengler

Last updated by author(s): Sep 28, 2023

Reporting Summary

Nature Portfolio wishes to improve the reproducibility of the work that we publish. This form provides structure for consistency and transparency in reporting. For further information on Nature Portfolio policies, see our Editorial Policies and the Editorial Policy Checklist.

Statistics

For all statistical analyses, confirm that the following items are present in the figure legend, table legend, main text, or Methods section.

n/a	Confirmed	
☐	☒	The exact sample size (*n*) for each experimental group/condition, given as a discrete number and unit of measurement
☐	☒	A statement on whether measurements were taken from distinct samples or whether the same sample was measured repeatedly
☐	☒	The statistical test(s) used AND whether they are one- or two-sided<br>*Only common tests should be described solely by name; describe more complex techniques in the Methods section.*
☒	☐	A description of all covariates tested
☒	☐	A description of any assumptions or corrections, such as tests of normality and adjustment for multiple comparisons
☐	☒	A full description of the statistical parameters including central tendency (e.g. means) or other basic estimates (e.g. regression coefficient) AND variation (e.g. standard deviation) or associated estimates of uncertainty (e.g. confidence intervals)
☐	☒	For null hypothesis testing, the test statistic (e.g. *F*, *t*, *r*) with confidence intervals, effect sizes, degrees of freedom and *P* value noted<br>*Give P values as exact values whenever suitable.*
☒	☐	For Bayesian analysis, information on the choice of priors and Markov chain Monte Carlo settings
☒	☐	For hierarchical and complex designs, identification of the appropriate level for tests and full reporting of outcomes
☒	☐	Estimates of effect sizes (e.g. Cohen's *d*, Pearson's *r*), indicating how they were calculated

Our web collection on statistics for biologists contains articles on many of the points above.

Software and code

Policy information about availability of computer code

Data collection	ImmunoSpot 7.0; BioTek Gen5 3.05; CFX Manager 3.1; xPONENT 3.1.971.0 (Luminex 200); Piccolo Xpress analyzer (Abaxis); FlowJo 10.9.0
Data analysis	All statistical analyses were conducted using GraphPad Prism version 9.1.2.

For manuscripts utilizing custom algorithms or software that are central to the research but not yet described in published literature, software must be made available to editors and reviewers. We strongly encourage code deposition in a community repository (e.g. GitHub). See the Nature Portfolio guidelines for submitting code & software for further information.

Data

Policy information about availability of data

All manuscripts must include a data availability statement. This statement should provide the following information, where applicable:

- Accession codes, unique identifiers, or web links for publicly available datasets
- A description of any restrictions on data availability
- For clinical datasets or third party data, please ensure that the statement adheres to our policy

All data are available in the manuscript or the supplementary material. Correspondence and requests for materials should be addressed to JRS (wsk7@cdc.gov).

Research involving human participants, their data, or biological material

Policy information about studies with human participants or human data. See also policy information about sex, gender (identity/presentation), and sexual orientation and race, ethnicity and racism.

Reporting on sex and gender	N/A
Reporting on race, ethnicity, or other socially relevant groupings	N/A
Population characteristics	N/A
Recruitment	N/A
Ethics oversight	N/A

Note that full information on the approval of the study protocol must also be provided in the manuscript.

Field-specific reporting

Please select the one below that is the best fit for your research. If you are not sure, read the appropriate sections before making your selection.

☒ Life sciences ☐ Behavioural & social sciences ☐ Ecological, evolutionary & environmental sciences

For a reference copy of the document with all sections, see nature.com/documents/nr-reporting-summary-flat.pdf

Life sciences study design

All studies must disclose on these points even when the disclosure is negative.

Sample size	As mouse studies were exploratory in nature, and effect size and standard deviation were not know for power analysis, group sizes were determined based on experience with the disease and vaccine models (100% lethal and 0% lethal, respectively), and confirmed to be within the acceptable range of degrees of freedom (DF) using the “resource equation” approach.
Data exclusions	No data were excluded in the analyses.
Replication	Overall in vivo experiments were not repeated, however they were designed to include appropriately-sized groups so that all donwstream data are representative of multiple biological replicates.
Randomization	Mice were assigned to groups by age and sex (to ensure even distribution). Individuals within age/sex groups were selected at random for experimental groups.
Blinding	For mouse studies, investigators were not blinded to group allocation during data acquisition. Clinical data were objective (e.g. weights), or based on detailed scoring systems developed for the disease model, and applied uniformly to all experimental groups by experienced personnel.

Reporting for specific materials, systems and methods

We require information from authors about some types of materials, experimental systems and methods used in many studies. Here, indicate whether each material, system or method listed is relevant to your study. If you are not sure if a list item applies to your research, read the appropriate section before selecting a response.

Materials & experimental systems

n/a	Involved in the study	
☐	☒	Antibodies
☐	☒	Eukaryotic cell lines
☒	☐	Palaeontology and archaeology
☐	☒	Animals and other organisms
☒	☐	Clinical data
☒	☐	Dual use research of concern
☒	☐	Plants

Methods

n/a	Involved in the study	
☒	☐	ChIP-seq
☒	☐	Flow cytometry
☒	☐	MRI-based neuroimaging

Antibodies

Antibodies used	Polyclonal rabbit anti-NP CCHFV antibody (IBT, 04-0011); Goat anti-rabbit secondary antibody (Alexa Flour 488, Invitrogen, A-11008)
Validation	IBT reports validation of CCHF anti-NP primary antibody via 1) Western blot: reactivity to CCHFV infected Vero cell lysate and HEK293 cell lysate transfected with recombinant CCHFV N protein, and 2) ELISA.

Eukaryotic cell lines

Policy information about cell lines and Sex and Gender in Research

Cell line source(s)	BSRT7/5 cells were provided by K.K. Conzelmann, Ludwig-Maximilians-Universität, Munich, Germany. HuH-7 cells were obtained from APATH LLC. Expi293F cells were purchased from ThermoFisher (A14527).
Authentication	Validation or authentication (certificates of analysis) were provided by company or provider at the time of acquisition.
Mycoplasma contamination	All virus, vaccine, and cell line stocks were confirmed to be free of Mycoplasma through the use of MycoAlert PLUS detection kit (Lonza LT07) and/or next generation sequencing.
Commonly misidentified lines (See ICLAC register)	N/A

Animals and other research organisms

Policy information about studies involving animals; ARRIVE guidelines recommended for reporting animal research, and Sex and Gender in Research

Laboratory animals	Ifnar-/- mice (B6.129S2-Ifnar1tm1Agt/Mmjax; mixed gender, 4–7 weeks of age) purchased from the Jackson Laboratory
Wild animals	N/A
Reporting on sex	Experimental groups have equal representation of both sexes.
Field-collected samples	N/A
Ethics oversight	Animal studies were conducted in compliance with the Guide for the Care and Use of Laboratory Animals and approved by the CDC Institutional Animal Care and Use Committee (IACUC) (protocol 3034SPECMOUC). The CDC is fully accredited by AAALAC International.

Note that full information on the approval of the study protocol must also be provided in the manuscript.
